# FGF-2 promotes angiogenesis through a SRSF1/SRSF3/SRPK1-dependent axis that controls VEGFR1 splicing in endothelial cells

**DOI:** 10.1186/s12915-021-01103-3

**Published:** 2021-08-25

**Authors:** Tao Jia, Thibault Jacquet, Fabien Dalonneau, Pauline Coudert, Elisabeth Vaganay, Chloé Exbrayat-Héritier, Julien Vollaire, Véronique Josserand, Florence Ruggiero, Jean-Luc Coll, Béatrice Eymin

**Affiliations:** 1 Institute For Advanced Biosciences, INSERM U1209, CNRS UMR5309, Université Grenoble Alpes, Site Santé, Allée des Alpes, 38700 La Tronche, France; 2 Key Laboratory of Drug-Targeting and Drug Delivery System of the Education Ministry and Sichuan Province, Sichuan Engineering Laboratory for Plant-Sourced Drug and Sichuan Research Center for Drug Precision Industrial Technology, West China School of Pharmacy, Sichuan University, Chengdu, 610041 China; 3 Institut de Génomique Fonctionnelle de Lyon, ENS de Lyon, UMR CNRS 5242, Université Lyon 1, 46 Allée d’Italie, 69364 Lyon Cedex 07, France

**Keywords:** Angiogenesis/endothelial cells/fibroblast growth factor/VEGFR1/SR proteins

## Abstract

**Background:**

Angiogenesis is the process by which new blood vessels arise from pre-existing ones. Fibroblast growth factor-2 (FGF-2), a leading member of the FGF family of heparin-binding growth factors, contributes to normal as well as pathological angiogenesis. Pre-mRNA alternative splicing plays a key role in the regulation of cellular and tissular homeostasis and is highly controlled by splicing factors, including SRSFs. SRSFs belong to the SR protein family and are regulated by serine/threonine kinases such as SRPK1. Up to now, the role of SR proteins and their regulators in the biology of endothelial cells remains elusive, in particular upstream signals that control their expression.

**Results:**

By combining 2D endothelial cells cultures, 3D collagen sprouting assay, a model of angiogenesis in cellulose sponges in mice and a model of angiogenesis in zebrafish, we collectively show that FGF-2 promotes proliferation, survival, and sprouting of endothelial cells by activating a SRSF1/SRSF3/SRPK1-dependent axis. In vitro, we further demonstrate that this FGF-2-dependent signaling pathway controls VEGFR1 pre-mRNA splicing and leads to the generation of soluble VEGFR1 splice variants, in particular a sVEGFR1-ex12 which retains an alternative last exon, that contribute to FGF-2-mediated angiogenic functions. Finally, we show that sVEGFR1-ex12 mRNA level correlates with that of FGF-2/FGFR1 in squamous lung carcinoma patients and that sVEGFR1-ex12 is a poor prognosis marker in these patients.

**Conclusions:**

We demonstrate that FGF-2 promotes angiogenesis by activating a SRSF1/SRSF3/SRPK1 network that regulates VEGFR1 alternative splicing in endothelial cells, a process that could also contribute to lung tumor progression.

**Supplementary Information:**

The online version contains supplementary material available at 10.1186/s12915-021-01103-3.

## Background

Angiogenesis, the formation of capillaries from pre-existing blood vessels, occurs in a variety of physiological and pathological conditions, including embryonic development, wound healing, and tumor growth [1]. Basic fibroblast growth factor-2 (FGF-2) is the prototype member of a family of structurally related fibroblast growth factors (FGFs) [2]. FGFs act by binding to and activating their cognate tyrosine kinase receptors (fibroblast growth factor receptor, FGFRs), leading to receptor dimerization, trans-phosphorylation, and activation of downstream signaling cascades [2]. They exert their angiogenic functions through both paracrine- and autocrine-dependent mechanisms because endothelial cells and also other stromal and tumor cells secrete FGFs and/or express FGFRs on their surface [3, 4]. In cultured endothelial cells, FGF-2 induces an angiogenic phenotype consisting of increased proliferation, survival, migration, proteinase production, and expression of specific integrins [5, 6]. In addition, FGF-2 stimulates endothelial cell barrier integrity by controlling the formation of tight junctions expressing VE-cadherin and other junctional proteins [7, 8]. In vivo, FGF-2 exerts a potent pro-angiogenic effect in different experimental models, including the chicken embryo chorioallantoic membrane (CAM) [9], rabbit/mouse cornea [10], and murine subcutaneous matrigel plug assays [11]. In zebrafish, FGF-2 also affects vascular outgrowth and is required for the maintenance of blood vessel integrity, including vessel stabilisation and to some extent vessel sprouting [12]. FGF-1/FGF-2 double knockout mice display poor wound healing compared with normal control mice, thereby indicating a pivotal role of FGF/FGFR signaling in tissue repair and neovascularization following injury [13, 14]. Overall, these studies highlight a predominant role of FGF-2 during various angiogenic processes. However, whether/how FGF-2 regulates post-transcriptional events in endothelial cells to promote angiogenesis remains largely unknown.

Alternative splicing (AS) produces different mature transcripts (mRNAs) from a single primary pre-mRNA. It is now well admitted that more than 90% of human protein-encoding genes undergo AS giving rise to different protein isoforms with distinct structural and functional properties [15]. Global alterations of AS have been shown to occur in cancer cells, including deregulated splicing of critical regulators of tumor angiogenesis such as vascular endothelial growth factor A (VEGF-A) [16, 17]. Conversely, the contribution of AS in tumor microenvironnment and, in particular, in cancer vasculature is still poorly documented. AS decisions are modulated by a number of splicing regulatory factors that function in a coordinate manner inside the spliceosome to promote or inhibit the inclusion of specific exons/introns into the mature mRNA [18, 19]. Among them, SR (serine-rich/arginine) proteins (SRSFs) belong to a family of phylogenetically conserved, structurally related pre-mRNA splicing factors that are required for both constitutive and alternative splicing [20]. Owing to their C-terminal domain known as the RS domain and rich in alternating serine and arginine residues, SR proteins are highly regulated by phosphorylation, notably by the SR-phosphorylating kinases SRPK1 and SRPK2, that control their activity inside the spliceosome and their sub-cellular/nuclear localization [21, 22]. SR proteins expression is largely deregulated in cancer. As an example, we previously reported the upregulation of SRSF1, SRSF2, and SRPK1 proteins in lung cancer patients [23]. In addition, in normal epithelial cells and various cancer types, we and others demonstrated the role of SR proteins in the regulation of VEGF-A or VEGFR1 alternative splicing [16, 24–26] and SRPK1 was shown to enhance the production of the pro-angiogenic splice variants of VEGF-A [16, 27–29]. In contrast, up to now, only a few studies have investigated the role of SR or SRPK proteins in primary endothelial cells as well as identified upstream stimuli that control their expression in this context.

In this study, using in vitro approaches and in vivo models of angiogenesis, we show that FGF-2 promotes proliferation, survival, and sprouting of primary endothelial cells by activating a signaling pathway involving the SR proteins SRSF1 and SRSF3 as well as SRPK1. This is leading to the generation of soluble VEGFR1 splice variants deprived of VEGFR1 transmembrane and tyrosine kinase domains, and more particularly of sVEGFR1-i13 and sVEGFR1-ex12 that retain an alternative last exon (exon 12). Interestingly, in squamous lung carcinoma patients, we also report a correlation between FGF-2, FGFR1, and sVEGFR1-ex12 mRNA levels, with patients exhibiting high levels of sVEGFR1-ex12 usage value also presenting high FGF-2/FGFR1 mRNA levels, and we further highlight sVEGFR1-ex12 as an independent poor prognosis marker in these patients. As a whole, these data identify a cross-talk between FGF-2 and splicing of VEGFR1 involved in the control of normal and pathological angiogenesis.

## Results

### FGF-2-induced endothelial cell proliferation and survival correlates with increased expression of SRSF1, SRSF3, and SRPK1 proteins

**Fig. 1:**
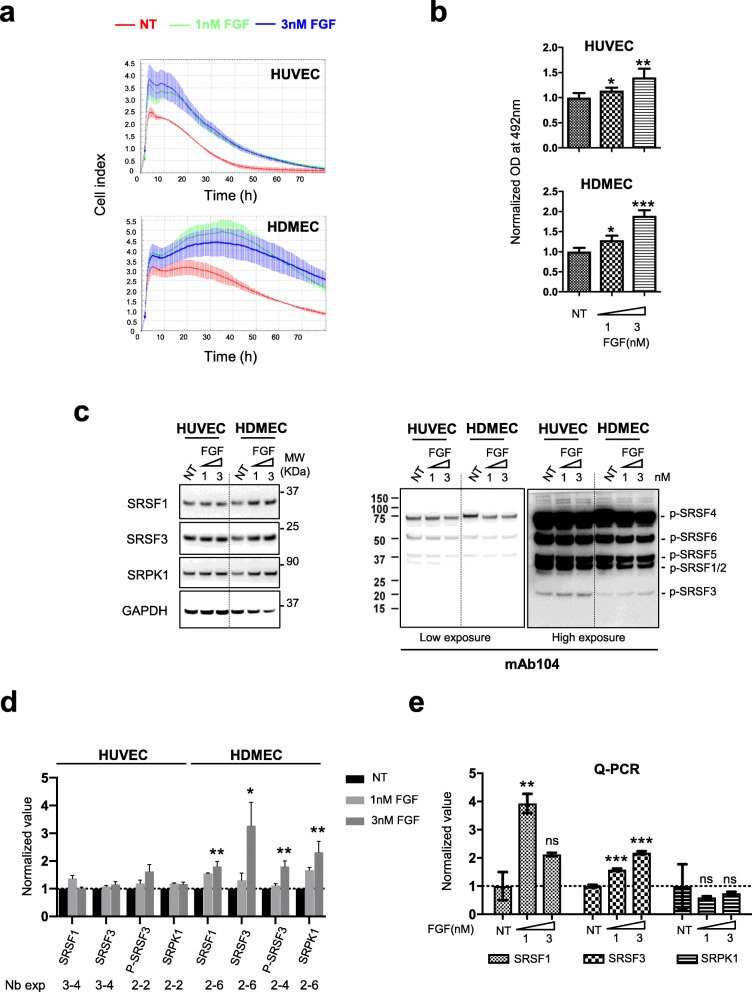
FGF-2 increases SRSF1, SRSF3 and SRPK1 protein levels in primary endothelial cells. **a**, **b** xCELLigence (**a**) or MTS (**b**) assay was used to assess HUVEC and HDMEC cellular adhesion, proliferation, and viability in response to FGF-2 at 1 or 3nM. Data are representative of at least 2 independent experiments performed in at least triplicate (mean ± SD, unpaired *t* test, **p<0.01; ***p<0.001). **c** Representative immunoblots for SRSF1, SRSF3, SRPK1 (left panel), and phospho-SR (p-SR) (mAb104, right panel, two exposure times) protein levels in HUVEC and HDMEC, respectively, treated with 1 or 3nM FGF-2 for 72 h. GAPDH was used as a loading control. NT: nontreated. **d** Semi-quantification using ImageJ software of SRSF1, SRSF3, P-SRSF3, or SRPK1 signal relative to GAPDH signal. Ratio obtained for NT group was arbitrarily assigned the value 1. Numbers below the graph indicate the number of biological replicates for each condition (mean ± SD, unpaired *t* test, *p<0.05, **p<0.01). **e** HDMEC were treated with 1 or 3nM FGF-2 as in (**c**). SRSF1, SRSF3, and SRPK1 mRNA levels were quantified by RT-qPCR in each condition. GAPDH was used as an internal control. Mean ± SEM are presented (*n*=4, unpaired *t* test, **p< 0.01, ***p<0.001, ns: not significant)

**Fig. 2:**
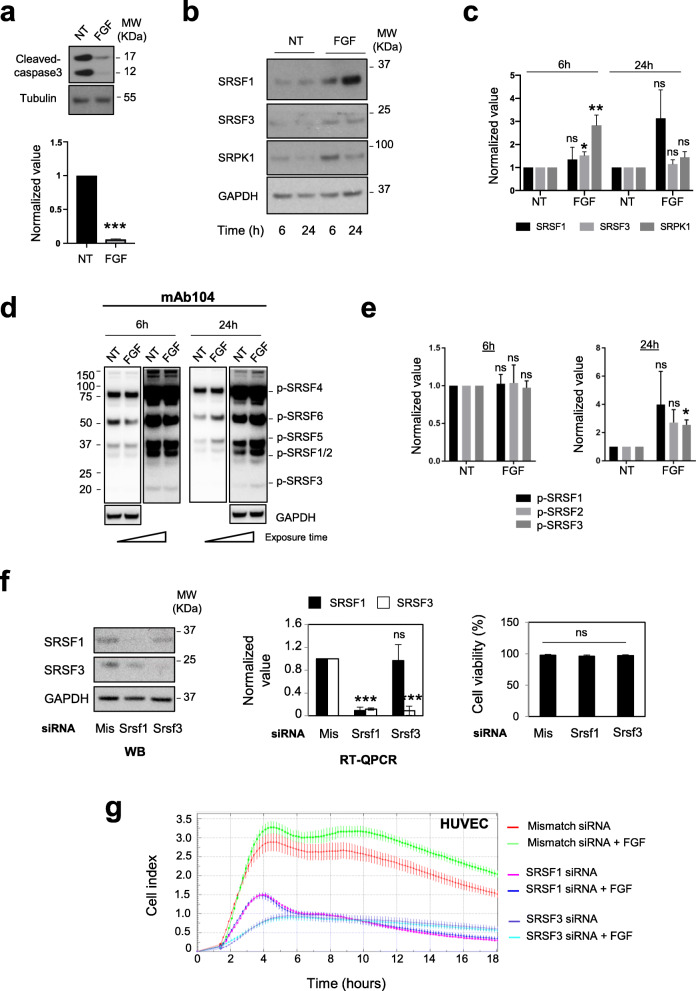
FGF-2-mediated HUVEC survival requires SR proteins accumulation and phosphorylation. **a** HUVEC were plated for 24 h in full medium, then cultured for 6 additional hours in EBM-2 basal medium supplemented (FGF) or not (-FGF/NT) with 3nM FGF-2 as indicated. *Upper panel:* immunoblot for cleaved-caspase 3. Tubulin was used as a loading control. *Lower panel:* semi-quantification of cleaved-caspase 3 signal relative to tubulin signals versus the values of the NT group by ImageJ (*n*=3, unpaired *t* test, ***p<0.001). **b**, **d** Immunoblots for SRSF1, SRSF3, and SRPK1 (**b**) or phospho-SR (**d**) proteins in HUVEC treated (FGF) or not (NT) with 3nM FGF-2 for 6 or 24 h as in (**a**). GAPDH was used as a loading control. **c**, **e** Semi-quantification by ImageJ of the indicated proteins signals relative to GAPDH signal. The ratio obtained for the NT group was arbitrarily assigned the value 1 (c, *n*=4; e, *n*=3; unpaired *t* test, *p<0.05, **p<0.01, ns: not significant). **f** *Left and middle panels:* representative immunoblots of SRSF1 and SRSF3 protein levels (left) or RT-qPCR analyses of *Srsf1* and *Srsf3* mRNA levels (middle) in HUVEC transfected during 48 h with either control (Mis), SRSF1 (Srsf1), or SRSF3 (Srsf3) siRNA as indicated. A 50:50 mixture of two distinct SRSF1 or SRSF3 siRNAs was used (*n*=4; unpaired *t* test, ***p<0.001, ns: not significant). *Right panel:* cell viability quantified by using trypan blue exclusion counting in HUVEC just before plating for xCELLigence assay. Mean ± SD are presented (siRNA group mismatch, *n*=3; siRNA group Srsf1, *n*=2; siRNA group Srsf3, *n*=3). **g** xCELLigence assay on HUVEC transfected with the indicated siRNA and treated or not with 3nM FGF-2 (*n*=3)

In order to identify new molecular mechanisms by which FGF-2 promotes angiogenesis, we used two primary human endothelial cell models, namely human umbilical vein (HUVEC) and human dermal microvascular endothelial cells (HDMEC), grown in standard 2 dimension cell cultures. We monitored cell adhesion, proliferation, and viability during 3 days in the presence of EBM-2 (endothelial cells basal medium-2) supplemented or not with 1 or 3nM FGF-2, using an xCELLigence system. In this assay, FGF-2 stimulated cell proliferation and/or prevented the death usually occuring in no serum conditions between 10 and 20 h after HUVEC or HDMEC plating (Fig. 1a). This FGF-2 protective effect was confirmed in an MTS assay (Fig. 1b). We previously identified members of the SR proteins family of splicing factors as critical regulators of VEGF-A alternative splicing in lung cancer cells [25, 26, 30]. We thus investigated the role of SR proteins in FGF-2 effects on endothelial cells. We observed a dose-dependent increase of SRSF1, SRSF3, and SRPK1 protein levels in HDMEC but not HUVEC treated with FGF-2 for 3 days (Fig. 1, c, d). This was associated with increased *Srsf1* and *Srsf3* mRNA levels (Fig. 1e). Using mAb104, a specific antibody targeting phospho-epitope on SR proteins, we further showed an accumulation of P-SRSF3 protein in HDMEC treated with FGF-2 (Fig. 1, c, d). Since the protective effects of FGF-2 were more significant in HDMEC than in HUVEC in this experimental design (Fig. 1, a, b), we set-up another experiment on HUVEC by analyzing the effects of FGF-2 at earlier stages (i.e., after 6 and 24 h treatment). In this experiment, FGF-2 significantly prevented apoptosis of HUVEC grown in no serum conditions, as detected by the inhibition of caspase-3 activation (Fig. 2a). In addition, in FGF-2-treated cells, increased expression of SRSF3 and SRPK1 proteins was observed at 6 h (Fig. 2, b, c), followed at 24 h by increased phosphorylation of various SR proteins, including SRSF3 (Fig. 2, d, e). To go further, SRSF1 or SRSF3 expression was neutralized in HUVEC by using siRNAs. The knockdown of either SRSF1 or SRSF3, as assessed by immunoblotting (Fig. 2f, left panel) and RT-qPCR (Fig. 2f, middle panel), did not significantly impact HUVEC viability as quantified by trypan blue exclusion before plating (Fig. 2f, right panel). However, using xCELLigence assay, we showed that HUVEC deprived of SRSF1 or SRSF3 do not respond to FGF-2 stimulation compared to HUVEC transfected with a control siRNA (Fig. 2g). In HUVEC, we also noticed that the knockdown of SRSF1 decreases SRSF3 protein and mRNA levels (Fig. 2f, left and middle panels), thereby suggesting the existence of regulatory cross-talks between SRSF1 and SRSF3 in these cells. Overall, these results demonstrated that FGF-2-induced endothelial cells proliferation and survival correlates with the activation of an SRSF1/SRSF3-dependent axis. After cell plating, we also observed a decreased adhesion, proliferation, and survival of untreated HUVEC deprived of SRSF1 or SRSF3 compared to HUVEC transfected with a control siRNA (Fig. 2g). These data suggest a more global role of SRSF1 and SRSF3 proteins in the biology of endothelial cells beyond FGF-2 response.

### FGF-2 requires SRPK1 activity to promote endothelial cells survival

**Fig. 3:**
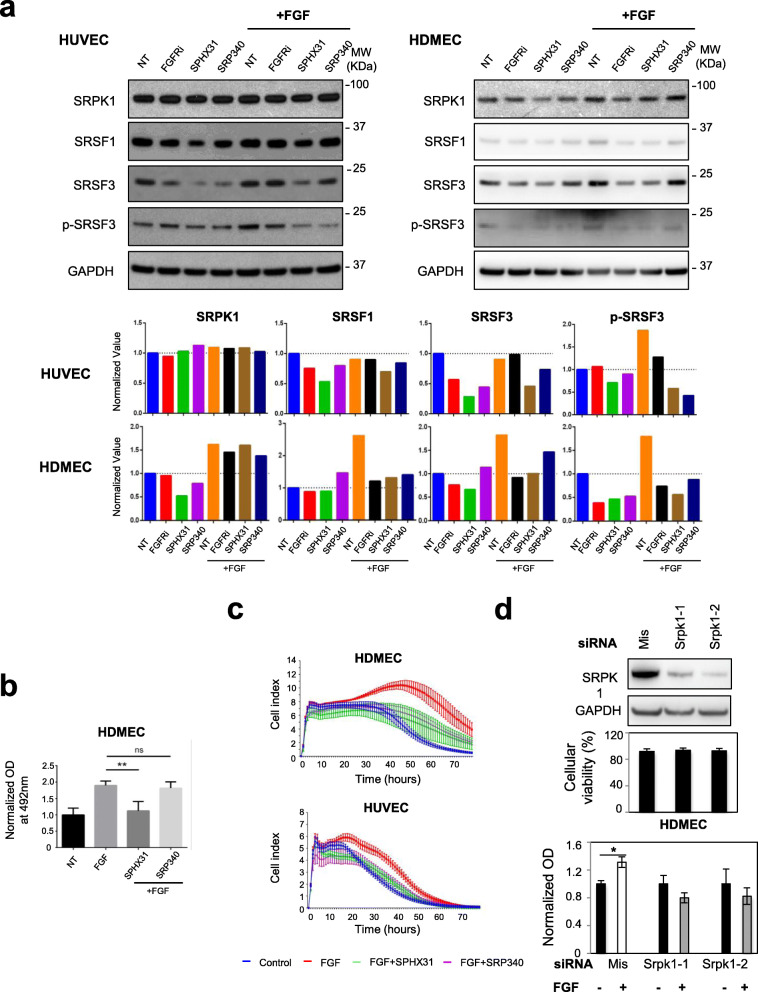
FGF2-mediated endothelial cells proliferation and survival requires SRPK1 activity. **a** *Upper panel:* immunoblots of the indicated proteins in HUVEC (left) and HDMEC (right) treated or not (NT) for 72 h with 10nM AZD4547 (FGFRi), 5μM SPHINX31 (SPHX31), or 10μM SRPIN340 (SRP340) in the presence or absence of 3nM FGF-2 as indicated. GAPDH was used as a loading control. Data representative of 2 (HUVEC) and 3 (HDMEC) independent experiments are presented. *Lower panel:* semi-quantification using ImageJ software of the signals obtained for the indicated proteins relative to GAPDH signal. The ratio obtained for the NT group was arbitrarily assigned the value 1. **b** MTS test was used to quantify HDMEC cellular viability in response to 72 h treatment with or without 3nM FGF-2 in the presence or absence of 5μM SPHINX31 (SPHX31) or 10μM SRPIN340 (SRP340) (*n*=4 technical replicates, unpaired *t* test, **p<0.01, ns: not significant). **c** xCELLigence assay was used to assess HUVEC and HDMEC cell adhesion, proliferation, and viability in response to 3nM FGF-2 with or without 5μM SPHINX31 (SPX31) or 10μM SRPIN340 (SRP340) (*n*=3). **d** HDMEC was transfected for 48 h with either mismatch (mis) or distinct SRPK1 siRNAs as indicated. *Upper panel:* representative immunoblot of SRPK1 knockdown. GAPDH was used as a loading control (*n*=3). *Middle panel:* cellular viability (%) was assessed after trypan blue staining in transfected HDMEC just before plating (*n*=5). *Lower panel:* MTS assay was used to quantify cellular viability in HDMEC deprived of SRPK1 (Srpk1) or not (Mis) and treated (+) or not (−) with 3nM FGF-2 for 48 h. Mean ± SD (*n*=3; unpaired *t* test, *p<0.05)

The observation that FGF-2 induces an accumulation of SRPK1 and/or phospho-SR proteins in endothelial cells (Figs. 1 and 2) prompted us to analyze the effects of ATP-competitive pharmacological inhibitors of SRPKs. To this end, HUVEC or HDMEC was treated with either SRPIN340, a potent selective SRPK1/2 inhibitor [28, 31] or SPHINX31, a selective SRPK1 inhibitor [29], in the presence or absence of 3nM FGF-2 during 72 h (Fig 3a). When used alone, SPHINX31 and/or SRPIN340 decreased SRSF1 and SRSF3, or SRPK1, SRSF3, and P-SRSF3 protein levels in HUVEC or HDMEC, respectively (Fig. 3a). Interestingly, similar variations were observed in response to AZD4547, a highly selective inhibitor of FGFR1/2/3 receptors (Fig. 3a). In addition, SPHINX31 and to a lower extend SRPIN340 prevented FGF-2-mediated increase of SRSF1, SRSF3, and P-SRSF3 protein levels in HDMEC, while SPHINX31 and SRPIN340 mainly reversed P-SRSF3 accumulation in the presence of FGF-2 in HUVEC (Fig. 3a). As a whole, these results indicated that a FGF-2/FGFR-dependent signaling pathway regulates SR proteins expression level and phosphorylation in primary endothelial cells. At the functional level, SPHINX31 reversed the protective effects of FGF-2 on endothelial cells, as quantified by MTS assay in HDMEC (Fig. 3b) and xCELLigence experiment in HDMEC and HUVEC (Fig. 3c). In addition, FGF-2 did not exhibit any protective effects in HDMEC deprived of SRPK1 compared to control cells (Fig. 3d). Of note, the knockdown of SRPK1 did not significantly affect cell viability before plating (Fig. 3d). These results showed that SRPK1 activity is required for FGF-2-mediated proliferative and protective effects in endothelial cells.

In normal epithelial cells as well as in cancer cells, it has been previously shown that inhibition of SRPKs switches VEGF-A pre-mRNA splicing towards anti-angiogenic VEGF-A splice variants, in particular VEGF_165_b [27–29]. As shown by immunoblotting, VEGF_165_b protein level did not increase in HUVEC or HDMEC treated with FGF-2, whatever the presence or absence of SPHINX31 or SRPIN340, compared to FGF-2 treatment alone (Additional File 1: Fig S1a). Furthermore, when we analyzed total VEGF-A, VEGF_121_, VEGF_165_, and VEGF_189_ mRNA levels by RT-qPCR in HDMEC treated or not with FGF-2 in the presence of SRPIN340 or SPHINX31, an increase of total VEGF-A, VEGF_165_, and VEGF_189_ mRNA levels was detected in cells co-treated with FGF-2 and SPHINX31 or SRPIN340 compared to FGF-2 alone (Additional File 1: Fig S1b). Therefore, these data indicated that the blocking effects of SRPK inhibitors on FGF-2 pro-angiogenic functions in endothelial cells does not correlate with a switch of VEGF-A pre-mRNA splicing towards anti-angiogenic VEGF_165_b, at least in our in vitro settings.

### FGF-2 requires SRSF1, SRSF3, and SRPK1 activities to promote endothelial cells sprouting

**Fig. 4:**
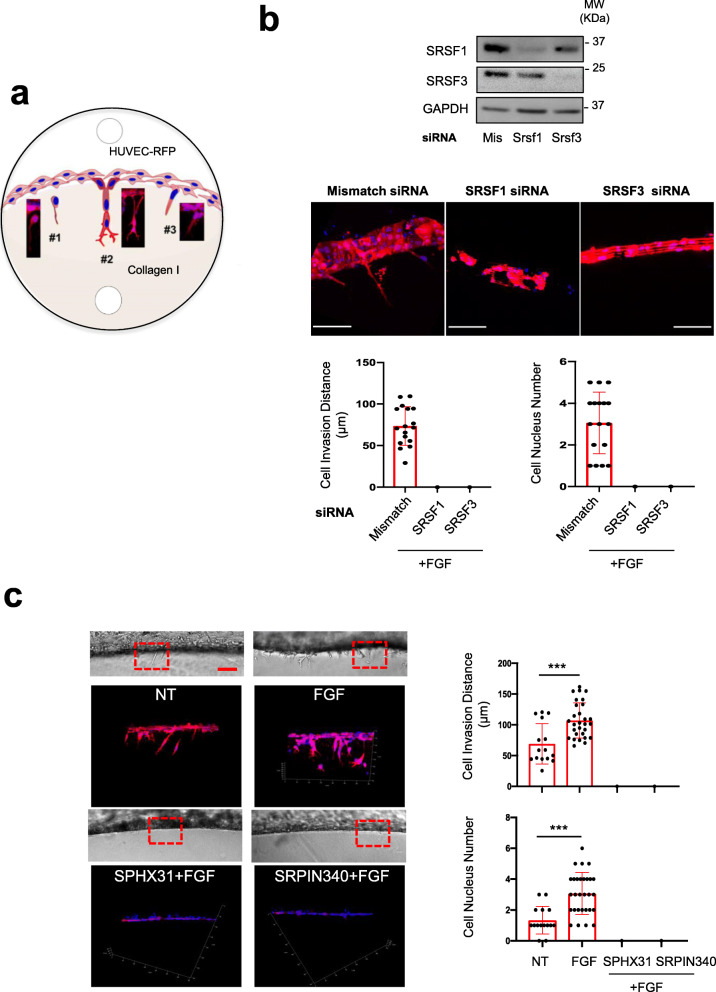
SRSF1/SRSF3 knockdown and SRPK1 inhibitors abrogate HUVEC-RFP invasion and sprouting in 3D collagen matrix. **a** Schematic representation of 3 different types of endothelial cell HUVEC-RFP behavior. #1: aborted endothelial cell sprouting, endothelial cells invade the collagen matrix without being followed by stalk cells; #2: capillary formation, complete process of endothelial cell invasion and sprouting. Filopodia projecting from the tip cells are observed; #3 early step of endothelial cell responses. For quantification, cell responses were scored according to these 3 types of cell behavior. **b** HUVEC-RFP were transfected for 48 h with control siRNA (mismatch) or a 50:50 mixture of two different SRSF1 (SRSF1) or SRSF3 (SRSF3) siRNAs before being analyzed in 3D invasion assay. *Upper panel:* immunoblots showing the efficient knockdown of SRSF1 or SRSF3 in HUVEC-RFP. GAPDH was used as a loading control. *Lower panels*: mosaic of confocal images covering around 300 μm of matrices border for different HUVEC-RFP post-transfection with indicated siRNA. HUVEC-RFP cells are in red and nuclei in blue. Scale bar = 100μm. Bar charts represent the quantifications of the invading distance of HUVEC-RFP within the 3D collagen matrices and the number of invading cells (nuclei) per 300 μm of gel border. Each black dot indicates one invading sprout. **c** HUVEC-RFP cells were treated or not (NT) with 3nM FGF-2 for 24 h in the presence or absence of 5μM SPHINX31 or 10μM SRPIN340 before performing 3D invasion assay. *Left top panels:* transmission light microscopy images showing invasive capacities of HUVEC-RFP within the 3D collagen gels. *Left bottom panels:* mosaic of confocal images covering around 300 μm of matrices border. HUVEC-RFP cells are in red and nuclei in blue. Scale bar = 100μm. *Right panel:* Bar charts represent the quantification of the invading distance of HUVEC- RFP within the 3D collagen matrices (top) and the number of invading cells (nuclei) per 300 μm of gel border (bottom). Each black dot indicates one invading sprout. **b**, **c** Graphs represent mean values ± SD of three independent experiments. Unpaired *t* test, ***p<0.001

Besides increased proliferation and survival, angiogenesis also requires migration and sprouting of endothelial cells. To further investigate the impact of the SRSF1/SRSF3/SRPK1 axis on FGF-2 pro-invasive functions, we used a three-dimensional (3D) in vitro system where Red Fluorescent Protein (RFP)-expressing HUVEC (HUVEC-RFP) were seeded on the surface of polymerized type I collagen gels as previously described [32]. In this experimental design, endothelial cells respond to angiogenesis inducers by developing invading sprouts (Fig. 4a). FGF-2 mediated sprouting was completely abrogated in HUVEC-RFP knockeddown for SRSF1 or SRSF3 compared to cells transfected with control (mismatch) siRNA, as indicated by measurements of the invasion distance and the number of nuclei in each sprout (Fig. 4b). Similar blocking effects were observed when HUVEC-RFP cells were treated with FGF-2 in the presence of either SPHINX31 or SRPIN340 (Fig. 4c). Collectively, these results indicated that FGF-2 requires SRSF1, SRSF3, and SRPK1 activities to promote endothelial cells sprouting.

### FGF-2-induced neo-angiogenesis in sponges engrafted in mice correlates with accumulation of SRSF1, SRSF3, and SRPK1 proteins in neo-vessels

**Fig. 5:**
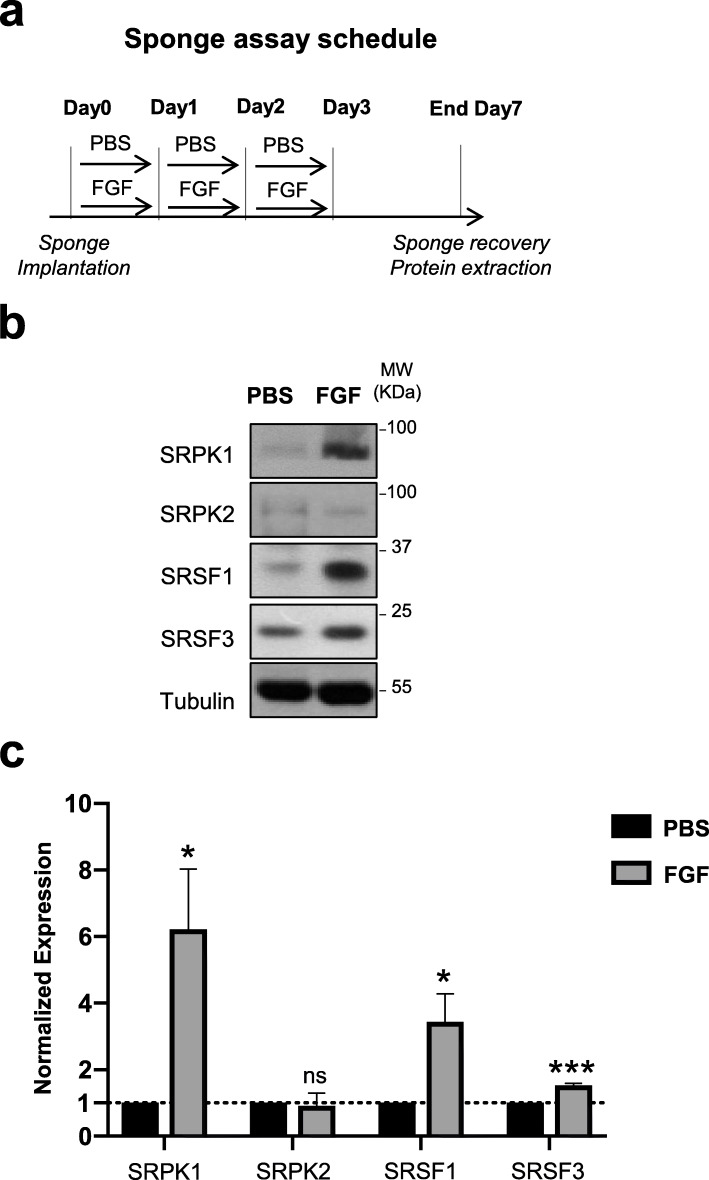
Endothelial cells accumulate SRSF1/3 and SRPK1 proteins upon FGF-2 treatment in an in vivo sponge assay. **a** Cellulose sponges loaded with FGF-2 or with PBS were engrafted under the skin of mice (*n*=6/group). As indicated in the graph, cellulose sponges were repeatedly injected. After 7 days, the sponges were collected and lysed for total protein extraction. **b** Representative immunoblots of SRPK1, SRPK2, SRSF1, and SRSF3 proteins from sponges treated with PBS or FGF-2. Tubulin was used as a loading control. **c** Semi-quantification using ImageJ software of FGF-2 effects on the indicated proteins relative to tubulin signals. The ratio obtained in PBS condition was arbitrarily assessed the value 1. Graphs represent mean values ± SD of 3 independent protein extracts (*t* test, *p<0.05, ***p<0.001, ns: not significant)

In order to investigate whether FGF-2 regulates SRSF1, SRSF3, or SRPK1 proteins in neo-angiogenic blood vessels in mice, we used a sponge assay as previously described [33]. The mice were subcutaneously engrafted 7 days before with a cellulose sponge loaded with FGF-2 (200 ng) or PBS as a control (Fig. 5a). In these conditions, FGF-2 acts as a chemo-attractant that potentiates the formation of neo-blood vessels around the sponge. Interestingly, when total proteins were extracted from control PBS- or FGF-2-treated sponges, we observed an accumulation of SRSF1, SRSF3, and SRPK1 proteins in sponges having received FGF-2 compared to PBS sponges (Fig. 5, b, c). Such increase was not detected with SRPK2 protein. These results highly suggest that the SRSF1/SRSF3/SRPK1 axis contributes to neo-angiogenesis mediated by FGF-2 in this sponge murine model.

### SRPK1 activity is required for vascular outgrowth in zebrafish

**Fig. 6:**
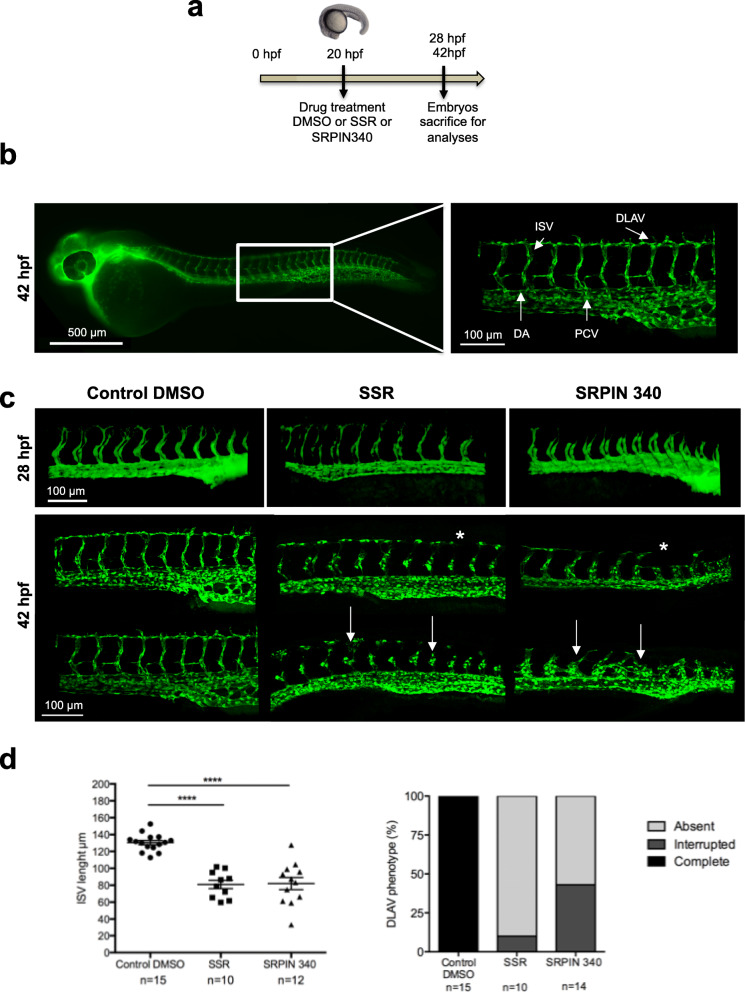
SRPIN340 treatment perturbs intersegmental vessels (ISV) sprouting and DLAV formation in zebrafish embryos. **a** The schedule sketch indicates the protocol used for drug treatment of Tg(fli1:EGFP) Casper embryos. **b** Immunofluorescence image of the vasculature of a Tg(fli1:EGFP) embryo at 42hpf (Left). Box represents the zoom area showing the trunk vasculature (Right). DLAV, dorsal longitudinal anastomic vessel; ISV, intersegmental vessel; DA, dorsal aorta; PCV, posterior cardinal vein. **c** Confocal images of EGFP^+^ vessels in the trunk of Tg(fli1:EGFP) zebrafish embryos at 28hpf (upper panel) and 42hpf (lower panel) after exposure to DMSO (as vehicle control), SSR or SRPIN 340 at 100μM. Arrows indicate disrupted ISVs. Stars point to incomplete DLAV formation. **d** Quantification of blood vessel formation defects. (Left) Quantification of ISV length with ImageJ software. Each value corresponds to the mean of length measurement of at least ten ISVs in the trunk of a same embryo. * represents a significant statistical difference between the indicated groups (one-way ANOVA test; ****p<0.0001). (Right) Quantification of DLAV phenotype scored as absent, interrupted, or complete in 42 hpf Tg(fli1:EGFP) embryos treated or not with SSR or SRPIN 340. Values are expressed in percentage of the total embryos analyzed in each condition. *n* = number of embryos



The development of the trunk vasculature in zebrafish is ideal for studying the impact of inhibitors on angiogenesis. Specifically, the dorsoventrally aligned intersegmental vessels (ISV) are believed to form via angiogenesis. ISVs sprout from the dorsal aorta (DA) at around 20 h post-fertilization (hpf), traverse in between the somites, and join to form the dorsal longitudinal anastomotic vessel (DLAV) (Fig. 6, a, b). In order to assess whether SRPK1 could also play a role in blood vessel formation in this model, we took advantage of the Tg(fli1:EGFP) Casper transparent zebrafish transgenic line in which the vasculature of the trunk can be followed by monitoring GFP-positive signal in developping optically clear embryos (Fig. 6, a, b). In these experiments, we used SRPIN340 instead of SPHINX31 because of the observed insolubility of SPHINX31 at the zebrafish raising temperature (28°C). The allosteric multi-FGFR blocker SSR128129E (SSR) was used as a positive control. This molecule was already shown to inhibit FGF signaling in zebrafish vascular development [12]. Exposure of developing embryos to 100 μM SRPIN340 or SSR started at 20 hpf to bypass effects of the chemicals on early embryonic development and stopped at 42hpf after completion of the primary angiogenic network formation (Fig. 6, a, b). At 28 hpf, phenotypic analysis revealed that ISVs in embryos treated with SSR or SRPIN340 have normally sprouted from the DA between the myotomes and that sprouts navigated through their stereotype ventrodorsal trajectory as in control embryos. Embryos treated with SRPIN340 showed nevertheless perceptible reduced or delayed ISV formation (Fig. 6c, Additional file 3: Video S1, Additional file 4: Video S2 and Additional file 5: Video S3). At 42 hpf, the ISVs in control embryos have reached the level of the dorsal neural tube and branched to form the DLAV (Fig. 6c and Additional file 6: Video S4). In contrast, embryos treated with SRPIN340 exhibited compromised ISVs formation and integrity (Fig. 6, c, d and Additional file 7: Video S5). ISVs displayed irregular shape and lengths with rounded-up endothelial cells indicative of defective endothelial cell–cell contacts and impaired tube formation (Fig. 6c). The DLAV was absent or appeared incompletely developed and displayed interruptions (Fig. 6, c, d). This vascular phenotype was reminiscent of the effects previously reported for SSR [12] and observed here in embryos treated in parallel with SSR (Fig. 6, c, d and Additional file 8: Video S6). As such, our data suggest that the FGF/SRPK1 axis is required for vascular outgrowth in zebrafish. Of note, when treated with SRPIN340, 42 hpf embryos also exhibited a pericardial edema (data not shown), a cardiotoxic phenotype that was already described for SSR [12].

**Additional file 3: Video S1.**


**Additional file 4: Video S2.**


**Additional file 5: Video S3.**


**Additional file 6: Video S4.**


**Additional file 7: Video S5.**


**Additional file 8: Video S6.**



### FGF-2 regulates VEGFR1 splicing in favor of soluble VEGFR1 splice variants through the SRSF1/SRSF3/SRPK1 signaling network

**Fig. 7:**
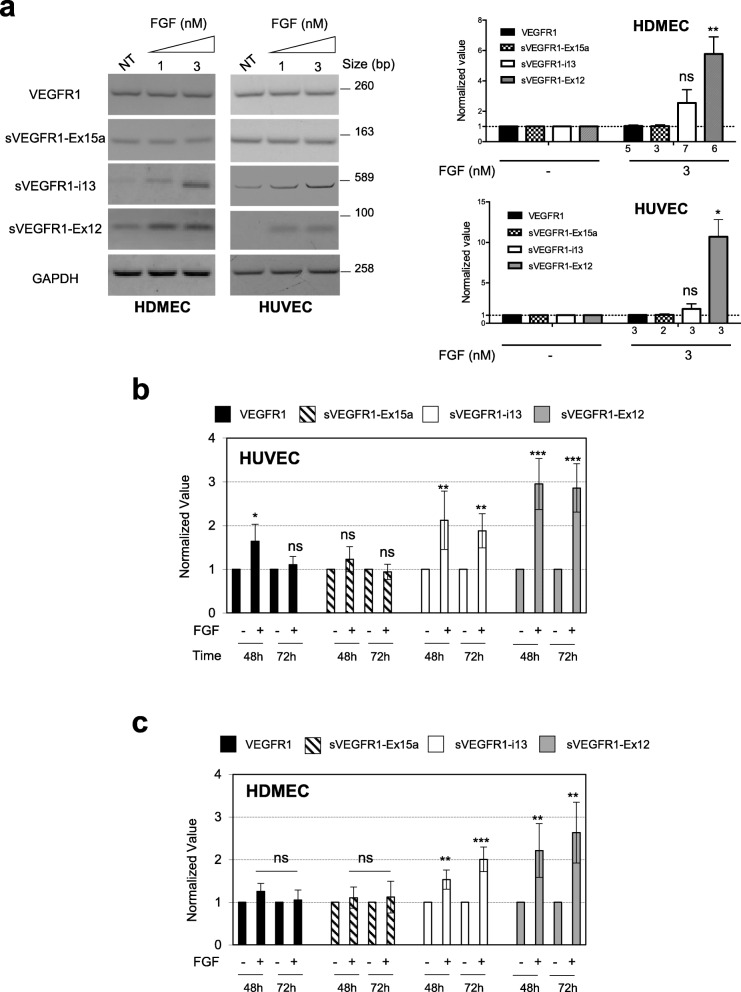
FGF-2 leads to the accumulation of sVEGFR1 splice variants in endothelial cells. **a** Dose effects of FGF-2 in HUVEC and HDMEC treated or not (NT) for 72 h with increasing concentrations of FGF-2. *Left panel:* representative RT-PCR analyses of VEGFR1 and sVEGFR1-ex15a, sVEGFR1-i13, and sVEGFR1-ex12 splice variants. GAPDH was used as an internal control. *Right panel:* semi-quantification using ImageJ software of PCR-specific signals related to GAPDH signal in 3nM FGF-2-treated cells. Ratio obtained in nontreated (NT) condition was arbitrarily assigned the value 1 (mean ± SD, unpaired *t* test, *p<0.05, **p<0.01, ns: not significant). Numbers below the graph indicate the number of biological replicates for each condition. **b**, **c** RT-qPCR analyses of VEGFR1 (black bars), sVEGFR1-ex15 (hatched bars), sVEGFR1-i13 (white bars), and sVEGFR1-ex12 (gray bars) mRNA levels in HUVEC (**b**) or HDMEC (**c**) treated or not with 3nM FGF-2 for 48 or 72 h as indicated. GAPDH was used as an internal control. Mean ± SD are presented (*n*=6; 2 technical replicates of 3 biological replicates excepted for VEGFR-1 and sVEGFR1-ex15 at 48 h *n*=4; 2 technical replicates of 2 biological replicates, unpaired *t* test, *p<0.05, **p<0.01, ***p<0.001, ns: not significant)

**Fig. 8:**
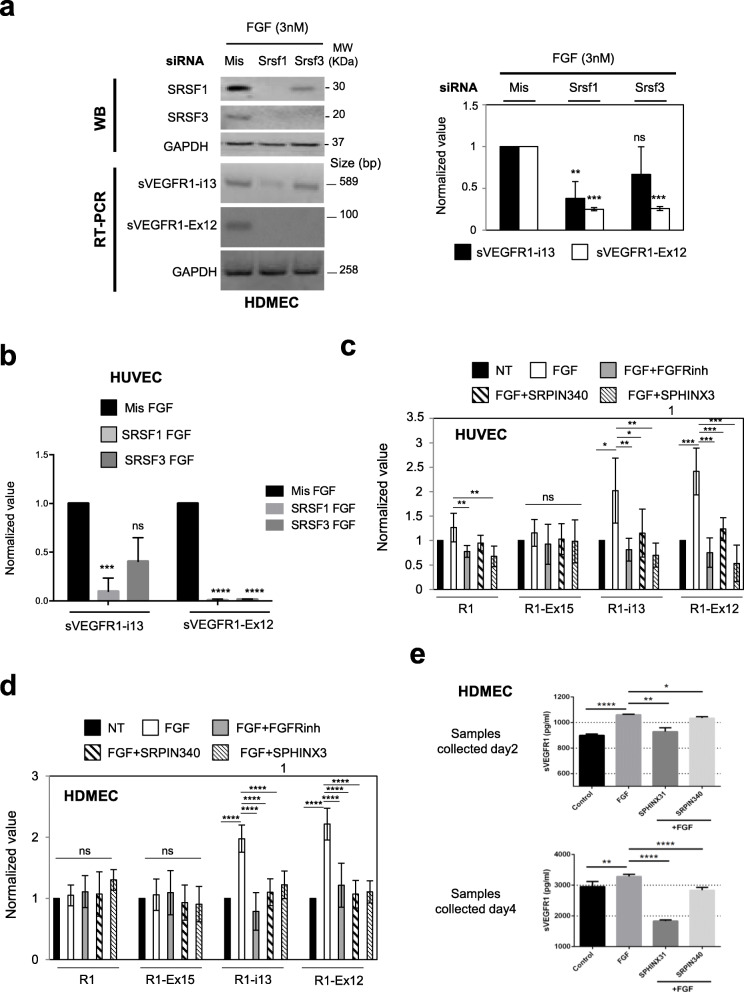
FGF-2 mediates accumulation of sVEGFR1 splice variants through a SRSF1/SRSF3/SRPK1-dependent axis. a HDMEC were transfected with control siRNA (mis) or specific SRSF1 (Srsf1) or SRSF3 (Srsf3) siRNA and treated with 3nM FGF-2 for 72 h. *Left panel:* representative immunoblots of SRSF1 or SRSF3 protein (WB) or PCR analyses (RT-PCR) of sVEGFR1-i13 and sVEGFR1-ex12 mRNA levels. GAPDH was used as a loading/internal control. *Right panel:* PCR data semi-quantification by ImageJ software. The sVEGFR1/GAPDH ratio obtained in control condition was arbitrarily assigned the value 1. Data are the mean ± SD of 3 and 4 biological replicates for sVEGFR1-ex12 and sVEGFR1-i13, respectively (unpaired *t* test, **p<0.01, ***p<0.001, ns: not significant). **b** sVEGFR1-i13 and sVEGFR1-ex12 mRNA levels were quantified by RT-qPCR in HUVEC transfected with either control siRNA (mis) or specific SRSF1 or SRSF3 siRNA and treated with 3nM FGF-2 for 48 h. GAPDH was used as an internal control. Mean ± SD are presented (*n*=3 biological replicates, unpaired *t* test, ***p<0.001, ****p<0.0001, ns: not significant). **c**, **d** VEGFR1 (R1), sVEGFR1-ex15 (R1-Ex15), sVEGFR1-i13 (R1-i13), and sVEGFR1-ex12 (R1-Ex12) mRNA levels were quantified by RT-qPCR in HUVEC (**c**) or HDMEC (**d**) treated (FGF, white bars) or not (NT, black bars) with 3nM FGF-2 for 48 h (HUVEC) or 72 h (HDMEC) in the presence or absence of 10nM AZD4547 (FGFRinh, gray bars), 10μM SRPIN340 (SRPIN340, black hatched bars), or 5μM SPHINX31 (SPHINX31, gray hatched bars). GAPDH was used as an internal control. Mean ± SD are presented (*n*=6; 2 technical replicates of 3 independent experiments, unpaired *t* test, *p<0.05, **p<0.01, ***p<0.001, ****p<0.0001, ns: not significant). **e** Quantification by ELISA assay of sVEGFR1 protein level in HDMEC supernatants collected 1 day or 3 days after treatment with or without (control) 3nM FGF-2 and 5μM SPHINX31 or 10μM SRPIN340. Data represent the mean ± SD of 3 (day 2) or 5 (day 4) independent experiments. *t* test, *p< 0.05, **p< 0.01, ***p<0.001, and **** *p*<0.0001

Having demonstrated a link between FGF-2 and splicing regulators in endothelial cells as well as unraveled the role of this cross-talk in various models of angiogenesis in vivo, we undertook experiments to identify target genes which splicing could be regulated by the FGF/SRSF/SRPK signaling network in endothelial cells. We recently reported that SRSF2 controls the splicing of VEGFR1 in lung cancer cells upon VEGF_165_ stimulation [26]. In addition, VEGF_165_ was previously shown to regulate VEGFR1 splicing in endothelial cells [34]. Therefore, we tested the possibility that FGF-2 also regulates VEGFR1 splicing. Different VEGFR1 mRNA splice variants have been reported to date that encode truncated soluble VEGFR1 (sVEGFR1) proteins with different C termini that are devoid of their transmembrane and tyrosine kinase domains (Additional File 2: Fig S2) [35, 36]. Three of them, namely sVEGFR1-i13 short, sVEGFR1-i13 long, and sVEGFR1-ex15a, predominantly contribute to the expression of sVEGFR1 proteins which circulate at high levels in patients with preterm preeclampsia [35]. sVEGFR1-i13 short and sVEGFR1-i13 long result from alternative polyadenylation at different sites in intron 13 to yield mRNAs encoding the same 867 amino acid sVEGFR1 protein isoform, but with either a 17 or 4146 nt 3′-UTR region (Additional File 2: Fig S2). sVEGFR1-ex15a results from activation of a cryptic 3′-splice acceptor site leading to the inclusion of an alternative last exon (exon 15). Another sVEGFR1 splice variant retaining an alternative last exon (exon 12) has been described but remains poorly studied. By combining RT-PCR (Fig. 7a) and/or RT-qPCR (Fig. 7b, c) experiments using specific primers for VEGFR1, sVEGFR1-ex15a, sVEGFR1-i13, and sVEGFR1-ex12, we showed that FGF-2 increases sVEGFR1-i13 and sVEGFR1-ex12 mRNA levels, but not sVEGFR1-ex15a mRNA level, in both HUVEC (Fig. 7a, b) and HDMEC (Fig. 7a, c). In addition, as detected by RT-PCR, the knockdown of SRSF1 by siRNA decreased the level of both sVEGFR1-i13 and sVEGFR1-ex12 mRNAs, while the knockdown of SRSF3 only significantly decreased that of sVEGFR1-ex12 in HDMEC treated with 3nM FGF-2 (Fig. 8a). Similar results were obtained in HUVEC as shown by RT-qPCR (Fig. 8b). Furthermore, in both HUVEC (Fig. 8c) and HDMEC (Fig. 8d), the inhibition of SRPK1 activity by using either SRPIN340 or SPHINX31 prevented FGF-2-induced increase of sVEGFR1-i13 and sVEGFR1-ex12 mRNA levels without affecting those of sVEGFR1-ex15a. Similar effects were obtained with AZD4547, the selective FGFR inhibitor. This was consistent with FGF/FGFR signaling regulating VEGFR1 splicing in endothelial cells. Of note, in HUVEC cells, AZD4547, SRPIN340 and SPHINX31 also reversed the slight increase of VEGFR1 mRNA level detected upon 48 h treatment (Fig. 7b and Fig. 8c), while no effect of these inhibitors on VEGFR1 mRNA level was observed in HDMEC (Fig. 8d). Importantly, the accumulation of sVEGFR1 transcripts in response to FGF-2 stimulation also correlated with increased levels of secreted sVEGFR1 proteins as quantified by ELISA in HDMEC supernatants (Fig. 8e). This increase was significantly reversed upon treatment with SPHINX31 or SRPIN340. In summary, in FGF-2-stimulated endothelial cells, VEGFR1 splicing is regulated by a mechanism involving SRSF1, SRSF3, and SRPK1 proteins leading to increased production of soluble VEGFR1 splice variants.

### sVEGFR1s contribute to FGF-2 pro-angiogenic functions in endothelial cells by a mechanism involving α5β1 integrin

**Fig. 9:**
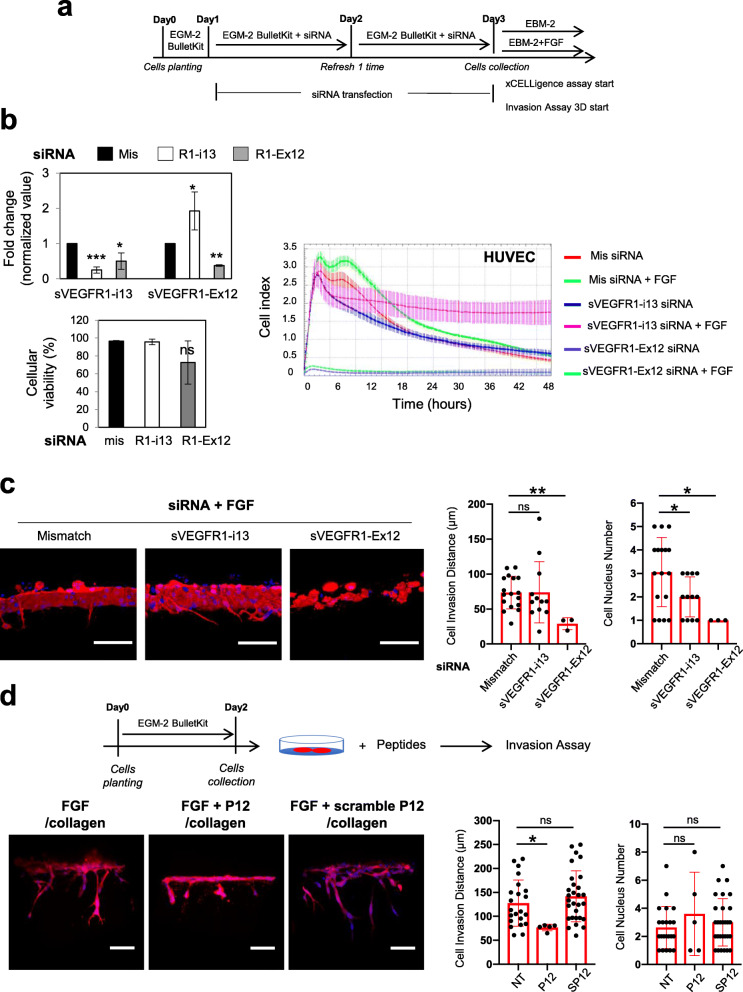
sVEGFR1-ex12 contributes to FGF-2 pro-angiogenic functions in endothelial cells. **a** The schedule sketch indicates the protocol used to generate sVEGFR1-i13 or sVEGFR1-ex12 knockeddown HUVEC-RFP cells by siRNA and to collect endothelial cells for xCELLigence (b) or 3D invasion (c) assay, respectively. **b** *Upper left panel:* sVEGFR1-i13 and sVEGFR1-ex12 mRNA levels were quantified by RT-qPCR in HUVEC-RFP transfected with either control siRNA (mis, black bars) or with a mixture (50:50) of two distinct siRNA against either sVEGFR1-i13 (white bars) or sVEGFR1-ex12 (grey bars). GAPDH was used as an internal control. Mean ± SD are presented (*n*=4; 2 technical replicates of 2 independent experiments, *t* test, *p<0.05, **p<0.01, ***p<0.001). *Lower left panel:* cellular viability quantified by using trypan blue exclusion counting in HUVEC-RFP just before plating for xCELLigence assay (mean ± SD, *n*=3; unpaired *t* test, ns: not significant). *Right panel:* xCELLigence assay was used to test FGF-2 (3nM) effects on adhesion/proliferation/survival of sVEGFR1-i13 or sVEGFR1-Ex12 depleted HUVEC-RFP (*n*=3). Mismatch siRNA was used as a positive control to ensure FGF-2 protective effects on cell viability in this experimental design. **c** 3D invasion assay in collagen I gels. *Left panels:* mosaic of confocal images covering around 300 μm of matrices border for HUVEC-RFP transfected with the indicated siRNA. HUVEC-RFP cells are in red and nuclei in blue. Scale bar = 100μm. Right panels: bar charts of the quantification of the invading distance of HUVEC-RFP within the 3D collagen matrices and the number of invading cells (nuclei) per 300 μm of gel border. Each black dot indicates one invading sprout. Graphs represent mean values ± SD (*n*=3; *t* test, *p<0.05, **p<0.01). **d** *Left panels:* confocal illustrations of the impact of the peptide p12 that blocks the interaction between sVEGFR1 and β1 integrin or a control scramble peptide on FGF-2-mediated HUVEC-RFP sprouting/invasion in collagen I gels (*n*=3). Scale bar is 100 μm. *Right panels:* Bar charts of the quantification of invading distance and the number of invading cells as indicated above (mean values ± SD, unpaired *t* test, *p<0.05, ns: not significant)

sVEGFR1 splice variants can serve as dominant-negative trapping proteins that inhibit the mitogenic effects of VEGF-A, as already described for the naturally produced VEGF-A antagonists [37]. However, it was also shown that sVEGFR1-i13 promotes endothelial cells adhesion and migration via its direct binding to α5β1 integrin [38]. In order to clarify the respective roles of sVEGFR1-i13 and sVEGFR1-ex12 splice variants in FGF-2-stimulated endothelial cells, HUVEC-RFP were transfected with control (mismatch), sVEGFR1-i13, or sVEGFR1-ex12 siRNA for 48 h (Fig. 9a) before performing xCELLigence (Fig. 9b) or 3D sprouting (Fig. 9c) assay. As detected by RT-qPCR, the knockdown of sVEGFR1-ex12 by siRNAs was accompanied by a decrease of sVEGFR1-i13 mRNA level (Fig. 9b). Conversely, sVEGFR1-i13 siRNAs, while strongly decreasing sVEGFR1-i13 mRNA level as expected, also led to an increase of sVEGFR1-ex12 mRNA level (Fig. 9b). This suggests the existence of feedback regulatory loops that control sVEGFR1 expression. In xCELLigence assay, we observed that HUVEC-RFP transfected with sVEGFR1-ex12 siRNAs do not attach nor proliferate and that FGF-2 does not exhibit any effects in these cells (Fig. 9b). In addition, sVEGFR1-ex12 siRNAs strongly decreased the stimulatory effects of FGF-2 on HUVEC-RFP sprouting (Fig. 9c). This was reminiscent of the effects observed upon knockdown of either SRSF1 or SRSF3 protein (Figs. 2g and 4b). Inversely, as quantified by XCelligence assay, transfection of HUVEC-RFP with sVEGFR1-i13 siRNAs did not prevent, rather prolonged, the protective effects of FGF-2 on HUVEC cultured in low serum condition (Fig. 9b) and had no significant inhibitory effects on FGF-2-stimulated sprouting although decreasing the number of endothelial cells in sprouts (Fig. 9c). This could be related to the upregulation of sVEGFR1-ex12 mRNA level detected in these cells (Fig. 9b). Taken together, these results are consistent with a more prominent role of sVEGFR1-ex12 in mediating FGF-2 pro-angiogenic functions. This was also supported by the observation that the knockdown of SRSF3, although not significantly affecting sVEGFR1-i13 mRNA level (Fig. 8a and b), strongly prevented FGF-2-mediated pro-angiogenic effects (Figs. 2g and 4b). A synthetic NYLTHRQ peptide (p12) that derives from a sequence localized inside the extracellular domain of all sVEGFR1 splice isoforms was previously shown to block the interaction between sVEGFR1 and α5β1 integrin [39]. Lastly, we showed that p12 but not a scramble peptide (sp12) inhibits FGF-2-mediated endothelial cells sprouting (Fig. 9d). These data indicate that the interaction between sVEGFR1s and α5β1 integrin on endothelial cells might play a role in FGF-2-induced endothelial cells sprouting.

### sVEGFR1-ex12/FGF-2/FGFR1 mRNA levels are correlated and sVEGFR1-ex12 is a poor prognosis marker in lung cancer patients

**Fig. 10:**
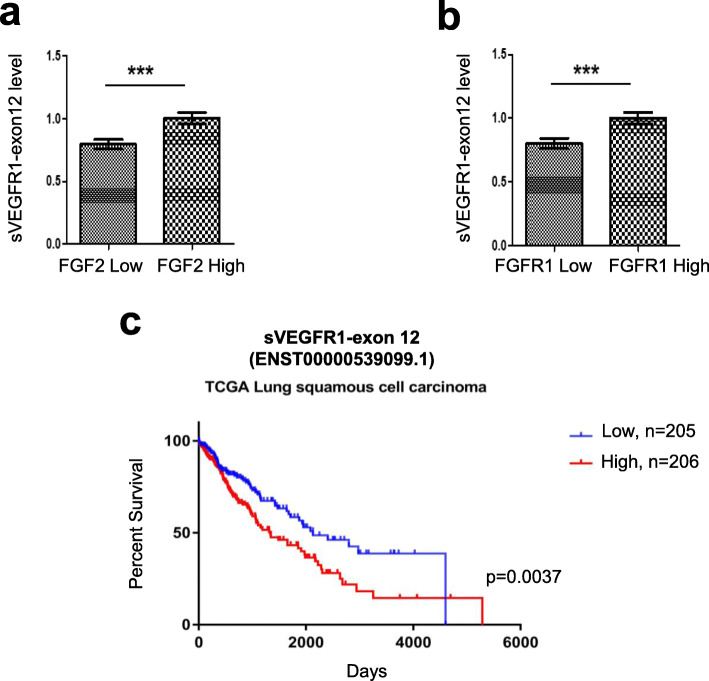
sVEGFR1-ex12 is a poor prognosis marker in squamous lung carcinoma patients. **a**, **b** Different sVEGFR1-exon12 usage value (exon expression value divided by gene expression value) between squamous lung carcinoma patients displaying low (<median, 1st and 2nd quartiles, *n*=243) and high (≥median, 3rd and 4th quartiles, *n*=244) FGF-2 (**a**) or FGFR1 (**b**) mRNA levels. Data is presented with mean ± SD. *p* values were calculated with an unpaired *t* test, ***p<0.001. **c** Overall survival analysis stratified according to sVEGFR1-exon12 usage value in squamous lung carcinoma patients for who clinical annotations were available (*n*=411). Low indicates the value below the median level (*n*=205) and high indicates values above the median level (*n*=206). The *p* value was calculated using a log-rank test

During tumor development, the FGF/FGFR network can be viewed as a dual network acting both on endothelial cells but also on tumor cells themselves through paracrine and autocrine functions [40]. It has been shown that the 8p12 locus (containing the FGFR1 gene) is frequently amplified in approximately 20% of squamous lung carcinoma [40]. To assess whether the link we identified in endothelial cells between FGF-2 signaling and sVEGFR1-ex12 could also be relevant in cancer cells, we took advantage of a publicly transcriptomic database, the TCGA research network initiated by NIH, including 411 squamous lung carcinoma patients with clinical annotations. In this cohort, we found a correlation between the mRNA level of either FGF-2 or FGFR1 and sVEGFR1-ex12 usage value, with patients displaying high FGF2 or FGFR1 mRNA level also exhibiting high sVEGFR1-ex12 level and inversely (Fig. 10, a, b). In addition, we found that patients with high sVEGFR1-ex12 levels display a worse prognosis compared to patients exhibiting low levels (Fig. 10c). Collectively, these results support a model in which the regulation of sVEGFR1-ex12 expression level by an FGF-2/FGFR1 axis could also contribute to the progression of squamous lung carcinoma.

## Discussion

**Fig. 11:**
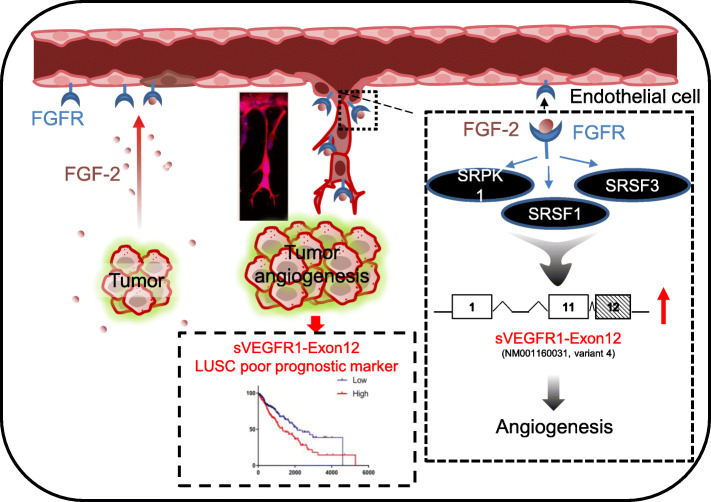
Graphical abstract for FGF-2-dependent regulation of sVEGFR1 splice variants in endothelial cells and its contribution to angiogenesis and lung tumorigenesis. In endothelial cells, FGF-2 stimulates a SRPK1/SRSF1/SRSF3 signaling pathway that controls VEGFR1 splicing in favor of sVEGFR1 splice variants, in particular sVEGFR1-ex12 (variant 4), that contribute to FGF-2 pro-angiogenic functions. In squamous lung carcinoma patients (LUSC), elevated sVEGFR1-ex12 usage value correlates with FGF-2/FGFR1 mRNA levels and with poor prognosis, thereby supporting a role of the FGF-2/FGFR1/sVEGFR1-ex12 signaling network in both physiological and pathological angiogenesis

Although much is known about vascular endothelial growth factor (VEGF)-dependent regulation of vascular development and angiogenesis, the role of fibroblast growth factors (FGFs) is less understood. In this study, by using in vitro 2D and 3D cultures as well as in vivo models of angiogenesis, we demonstrated that FGF-2 promotes proliferation, survival, and migration of endothelial cells by activating a SRSF1/SRSF3/SRPK1-dependent signaling network. This signaling regulates VEGFR1 splicing in favor of sVEGFR1 splice variants, in particular sVEGFR1-ex12 retaining an alternative last exon 12 (Fig. 11). Several lines of evidence exist showing FGF regulation of the VEGF system during the angiogenic process [4]. Hence, VEGF-A is an important downstream mediator of the mitogenic activity of FGF in endothelial and stromal cells [10, 41]. In addition, basal FGF stimulation of the endothelium is required for the maintenance of VEGFR2 expression and the ability of endothelial cells to respond to VEGF-A stimulation [7, 42]. Therefore, through the demonstration that FGF-2 also targets VEGFR1 in endothelial cells, our study adds a novel layer of complexity inside the cross-talks existing between FGF- and VEGF-A-dependent signaling pathways to control angiogenesis.

It has been previously shown that endothelial cells exhibit specific splicing programs that are distinct from those of epithelial and fibroblast cells [43]. Nevertheless, the molecular mechanisms that regulate splicing in endothelial cells remain largely unknown. In this study, by using both in vitro and in vivo models of angiogenesis, we showed that FGF-2 promotes angiogenesis by controlling the expression/activity of components of the alternative splicing machinery in endothelial cells. More specifically, we identified SRSF1, SRSF3, and SRPK1 proteins as critical downstream effectors of FGF-2 pro-angiogenic functions. In addition, we showed increased *Srsf1* and *Srsf3* mRNA levels in FGF-2-stimulated endothelial cells. Interestingly, SRPK1 and SRSF1 proteins were recently found to be highly expressed in lung tumor endothelium compared to normal lung endothelium and the upregulation of SRSF1 and SRPK1 was dependent on the transcription factor Wilms tumor suppressor 1 (WT1) [44]. Since functional links exist between WT1 and FGF-2 signaling pathways, it remains to be determined whether WT1 could play a role in FGF-2-induced SRSF1/SRSF3/SRPK1 expression in endothelial cells.

We also demonstrated that FGF-2 leads to the accumulation of P-SRSF3 protein and that pharmacological inhibition of SRPK1, by using either SRPIN340 or SPHINX31, prevents FGF-2-mediated angiogenesis which correlates with decreased level of P-SRSF3. Interestingly, one study previously demonstrated that Cdc2-like kinases (CLKs), another family of SR-phosphorylating kinases, are involved in alternative splicing of tissue factor in TNF-alpha stimulated endothelial cells and impact endothelium pro-coagulant activity [45]. Therefore, these and our results highly support a role of SR proteins phosphorylation in the biology of endothelial cells. Importantly, in Wilms (WT1) tumor cells, uveal and cutaneous melanoma cells as well as in normal podocytes and retinal pigmental epithelial cells, SRPIN340 or SPHINX31 has been previously shown to increase the expression of the anti-angiogenic VEGF_165_b splice variant [16, 27–29]. Similarly, in primary epithelial cells, SRSF1 was previously found to control VEGF-A splicing in favor of pro-angiogenic splice variants [16, 24]. In our in vitro studies performed in HUVEC and HDMEC, we did not see any reproducible variations of VEGF_165_b protein level upon treatment with FGF-2, whatever the presence or absence of SRPK1 inhibitors (Additional File 1: Fig S1a) or the knockdown of SRSF1/SRSF3 (data not shown). Although these in vitro data do not preclude that a switch of VEGF-A splicing in favor of anti-angiogenic splice variants could contribute to the negative effects of SRPK1 inhibitors or SRSF1/SRSF3 knockdown on FGF-2-mediated effects in vivo, they suggest that splicing patterns affected by SRSFs/SRPKs might vary depending on upstream stimuli as well as cell types (e.g., primary versus tumoral, epithelial versus endothelial).

We also identified VEGFR1 as a target of FGF-2 signaling pathway in endothelial cells. Hence, we showed that FGF-2 increases the mRNA level of sVEGFR1-i13 and sVEGFR1-ex12 splice variants, which correlates with enhanced expression of sVEGFR1 protein level in the supernatants of endothelial cells. We further demonstrated that SRSF1, SRSF3, and SRPK1 proteins are involved in FGF-2-induced sVEGFR1-ex12 expression. Interestingly, using the Exonic Splicing Enhancer (ESE) finder website (http://exon.cshl.edu/ESE) that allows to identify consensus RNA sequences bound by RNA Binding Proteins (RBP), we found a consensus ESE for SRSF1 inside exon 12 (score 2.26729), suggesting that SRSF1 binding could directly favor its inclusion in response to FGF-2 stimulation. At the functional level, we further demonstrated that the knockdown of sVEGFR1-ex12 strongly prevents FGF-2-mediated pro-angiogenic functions (e.g., increased survival and sprouting of endothelial cells). Of note, sVEGFR1-i13 mRNA level decreased in HUVEC transfected with sVEGFR1-ex12 siRNAs. This suggested that both sVEGFR1-i13 and sVEGFR1-ex12 splice variants contribute to FGF-2 pro-angiogenic functions. However, we also found that [1] sVEGFR1-i13 siRNAs, which strongly decrease sVEGFR1-i13 mRNA level but also increase sVEGFR1-ex12 mRNA level, does not prevent, rather enhances the protective effects of FGF-2 on HUVEC cultured in low serum condition (Fig. 9b) and has no significant inhibitory effects on FGF-2-stimulated sprouting (Fig. 9c) [2]; the knockdown of SRSF3, although not significantly affecting sVEGFR1-i13 mRNA level (Fig. 8a, b), strongly prevents FGF-2-dependent effects (Figs. 2g and 4b). As a whole, these results indicate that sVEGFR1-ex12 could play a more prominent role in mediating FGF-2 pro-angiogenic functions compared to sVEGFR1-i13.

sVEGFR1 splice variants were initially found to inhibit the mitogenic effects of VEGF-A by functioning as dominant-negative trapping proteins [37]. However, it was later on shown that local guidance of emerging vessel sprouts requires sVEGFR1 to support the formation of a VEGF-A gradient through sVEGFR1/VEGF-A interaction and local inactivation of VEGFR2 signaling [46, 47]. Moreover, it was further demonstrated that sVEGFR1-i13 is deposited by cultured endothelial cells in the extracellular matrix, where it determines endothelial cell adhesion and migration [38]. This activity is mediated by sVEGFR1-i13 interaction with the α5β1 integrin that shifts classical adhesion pathway to a more dynamic, motile phenotype of endothelial cells [48]. Interestingly, in our 3D collagen I invasion assay, we observed that a peptide that blocks specifically the interaction between sVEGFR1 and α5β1 integrin prevents FGF-2-induced endothelial cells sprouting. As a whole, these results support the idea that sVEGFR1 could contribute to FGF-2 pro-angiogenic functions by impacting on the local VEGF-A gradient around the sprouts as well as by interacting with α5β1 integrin on endothelial cells.

Besides its role in physiological context, angiogenesis is also essential for tumor growth and progression [1]. We finally report the existence of a correlation between the mRNA levels of either FGF-2 or FGFR1 and sVEGFR1-ex12 usage value in squamous lung carcinoma (SQLC) patients together with a worse prognosis in patients displaying elevated sVEGFR1-ex12 usage value. These data are in favor of a role of the FGF-2/FGFR1/sVEGFR1 cross-talk in lung tumor progression. FGF/FGFR signaling pathway is an attractive therapeutic target in SQLC owing to the frequent FGFR1 amplification in this histological subtype [40]. Consistently, various clinical trials using FGFR inhibitors as single-agent therapy, such as AZD4547, have been performed in SQLC patients with only modest response up to now, raising the possibility that therapeutic response could be enhanced with combination therapy [49, 50]. Based on our data, it could be therefore interesting to test whether inhibition of FGF/FGFR signaling pathway in combination with SRPK inhibitors could provide a benefit advantage in these patients. This could occur through decreased levels of sVEGFR1s and inhibition of tumor angiogenesis.

## Conclusions

In this study, we identify a signaling network by which FGF-2 exerts its pro-angiogenic functions that involves components of the splicing machinery, namely SRSF1, SRSF3, and SRPK1 proteins, together with sVEGFR1 splice variants. We also provide evidence of a correlation between high levels of FGF-2/FGFR1 and sVEGFR1-ex12 in squamous lung carcinoma patients. As a whole, this study adds a novel layer of complexity inside the cross-talks existing between FGF- and VEGF-A-dependent signaling pathways to control physiological and pathological angiogenesis.

## Methods

### Cell culture and reagents

Human umbilical vein endothelial cells (HUVEC; Lonza, France, cat#2519A) and red fluorescent protein expressing human umbilical vein endothelial cells (RFP-HUVEC) (obtained after direct retroviral infection of commercial HUVEC with an RFP expressing retrovirus) were cultured in full medium of EGM-2 BulletKit (Lonza, France, cat#CC-3162) including EBM-2 (endothelial cell basal medium-2; Lonza, France, cat#3156) and corresponding supplements (Lonza, France, cat#4176) optimized for endothelial cell culture. Experiments were performed with cells between passages 2 and 5. Human dermal microvascular endothelial cells (HDMEC; Lonza, France, cat#2516; passages 2 to 7) were cultured in full medium of EGM-2 MV BulletKit (Lonza, France, cat#3202) including EBM-2 and corresponding supplements. Cells were cultured on plates coated with 1.33μg/cm^2^ collagen I (Corning, cat#354236, Boulogne Billancourt, France) at 37°C in a humidified atmosphere containing 5% CO_2_. The Countess^TM^ automated cell counter (ThermoFisher) was used to determine cell count and viability (live, dead, and total cells) accurately and precisely, using the standard trypan blue technique. Only HUVEC/HDMEC with viability>95% was used for experiments. Basic fibroblast growth factor (FGF-2) was purchased from R&D systems (cat#233-FB/CF, Lille France). It was resuspended in sterile PBS 1X at a stock concentration of 100μg/ml, aliquoted, and stored at −20°C. AZD4547 and SRPIN340 were purchased from Selleckchem, dissolved in DMSO at a 10-mM stock solution, aliquoted, and stored at −80°C before the use at a final concentration of 10nM and 10μM, respectively. SPHINX31 was purchased from Axon Medchem (Groningen, The Netherlands) and was resuspended at a 10-mM stock solution in DMSO, aliquoted, and stored at −80°C. SPHINX31 was diluted at a working concentration of 5–10μM in full medium, dilution was vortexed and heated at 37°C just before use. In most of the in vitro experiments with HUVEC or HDMEC, full medium was replaced by EBM-2 (basal medium) at the time of the treatment with FGF-2 (plus or minus spliceosome inhibitors) in order to specifically analyze FGF-2 effects and to avoid confounding effects of the supplements contained in the EGM-2/EGM-2 MV Bulletkit. Of note, for RNA/protein collection in HUVEC treated during 48 or 72 h, 10% full medium in EBM-2 was used instead of basal medium EBM-2. The peptides p12 (NYLTHRQ) and scramble p12 (sp12, LTQNYRH) were from Covalab. They were resuspended in water at 10mg/ml and used at a final concentration of 10μg/ml.

### Transfection of siRNAs in primary endothelial cells

siRNA transfection was performed using lipofectamine RNAiMAX Reagent (Invitrogen) according to the manufacturer’s recommended protocol. Briefly, cells were firstly seeded at the density of 0.2–0.3 × 10^6^ cells/well/2mL in 6-well plate in full medium for 24 h. Two rounds of transfection (24 and 48 h after plating) were then performed before cells were plated in basal medium with/without FGF-2 and spliceosome inhibitors for xCELLigence analysis or sprouting assays. The siRNAs used in this study were from Eurogentec (Seraing, Belgium): mismatch: 5′-UCG-GCU-CUU-ACG-CAU-UCA-A-3′ (forward) and 5′-UUG-AAU-GCG-UAA-GAG-CCG-A-3′ (reverse); SRSF1-a: 5′-GAA-AGA-AGA-UAU-GAC-CUA-U-3′ (forward) and 5′-AUA-GGU-CAU-AUC-UUC-UUU-C-3′ (reverse); SRSF1-b: 5′-UAA-CUU-ACC-UCC-AGA-CAU-C-3′ (forward) and 5′-GAU-GUC-UGG-AGG-UAA-GUU-A-3′ (reverse); SRSF3-a: 5′-CGA-GAG-CUA-GAU-GGA-AGA-ACA-3′ (forward) and 5′-UGU-UCU-UCC-AUC-UAG-CUC-UCG-3′ (reverse); SRSF3-b: 5′-GAC-GGA-AUU-GGA-ACG-GGC-UUU-3′ (forward) and 5′-AAA-GCC-CGU-UCC-AAU-UCC-GUC-3′ (reverse); SRPK1-a: 5′-GCU-AAU-GAC-UGU-GAU-GUC-CAA-AA-3′ (forward) and 5′-UUU-UGG-ACA-UCA-CAG-UCA-UUA-GC-3′ (reverse); SRPK1-b: 5′-CCA-UGU-GAU-CCG-AAA-GUU-AGG-3′ (forward); 5′-UAA-CUU-UCG-GAU-CAC-AUG-GUA-3′ (reverse); sVEGFR1-i13-a: 5′-UAA-CAG-UUG-UCU-CAU-AUC-A-3′ (forward) and 5′-UGA-UAU-GAG-ACA-ACU-GUU-A-3′ (reverse); sVEGFR1-i13-b: 5′-UCU-CGG-AUC-UCC-AAA-UUU-A-3′ (forward) and 5′-UAA-AUU-UGG-AGA-UCC-GAG-A-3′ (reverse); sVEGFR1-ex12-a: 5′-CAA-GCU-UCU-CUU-CCA-ACU-ACU-3′ (forward) and 5′-UAG-UUG-GAA-GAG-AAG-CUU-GUA-3′ (reverse); sVEGFR1-ex12-b: 5′-CCA-GCU-AAC-AGU-UCU-UUC-AUG-3′ (forward) and 5′-UGA-AAG-AAC-UGU-UAG-CUG-GUG-3′ (reverse).

### xCELLigence assay

An xCELLigence Real-Time Cell Analysis assay (RTCA, ACEA Biosciences) was used to continuously monitor HUVEC and HDMEC cellular adhesion, viability, and proliferation. This method is based on the measurement of impedance to study cell-response profiling [51]. Before use, xCELLigence E-plates (8 wells/plate, 3 plates/experiment) were pre-heated at 37°C, washed once in PBS 1X, coated for 1 h at 37°C with 100μl/well of collagen I (0.1mg/ml in water), and washed three additional times in PBS 1X. In order to reduce evaporation, all empty wells and wells’ gap were filled with water. To study FGF-2 effects, 1.0 × 10^4^ cells/well of low passages HUVEC or HDMEC washed once in HANKs buffer to remove all traces of trypsin were seeded in EBM-2 basal medium containing or not 1/3nM FGF-2, and impedance was measured every 15 or 30 min during approximately 72 h. To study the effects of FGF-2 on HUVEC deprived of either SRSF1, SRSF3, sVEGFR1-i13, or sVEGFR1-ex12, cells were transfected with control or specific siRNA for 48 h then seeded in xCELLigence E-plates at a density of 2.5 × 10^4^ cells/well in EBM-2 basal medium containing or not 3nM FGF-2. Impedance was monitered every 5 or 10 min during approximately 24 or 48 h. To study the impact of splicesome inhibition on the effects of FGF-2, 2.5 × 10^4^ HUVEC or HDMEC cells per well were seeded in EBM-2 basal medium in the presence or absence of 3nM FGF-2 with or without either SPHINX31 (5μM) or SRPIN340 (10μM) and the impedance was measured every 30 min during approximately 72 h. All experiments were performed in at least triplicate wells for each condition.

### MTS cell viability assay

The cell viability was measured using the MTS-based cell viability assay in 96 well plates (Promega, Les Ulis, France). To test FGF-2 dosage effects, HUVEC or HDMEC was plated in full medium for 24 h, then EBM-2 basal medium was added and cells were treated or not with 1nM or 3nM FGF-2 for 72 additional hours. To study the impact of splicesome inhibitors on FGF-2 effects, a similar protocol was used with endothelial cells being treated or not with 3nM FGF-2 in the presence or absence of SRPK1/2 inhibitors SPHINX31 (5μM) or SRPIN340 (10μM). To test the impact of SRPK1 knockdown on cell viability, 0.25 × 10^6^ HDMEC cells/well were plated in 6-well plates in full medium for 24 h, subjected to two rounds of transfection in full medium as described above, then plated in EBM-2 basal medium and treated or not with 3nM FGF-2 for 48 additional hours. Four technical replicates were done per condition. After treatment, cells were washed with PBS 1X. Then, 10 μl of WST-1 reagent was added to each well. Plates were incubated at 37°C for 2 h. After the incubation period, plates were mixed gently on an orbital shaker for 1 min and the absorbance of each sample was measured at 492 nm using Beckman Coulter AD 340s (Fullerton, CA, USA).

### Antibodies and immunoblotting

Antibodies used in this study were SRSF1 (Invitrogen, cat#32-4500; 1:1000 dilution), SRSF3 (Invitrogen, cat# 33-4200; 1:1000 dilution), SRPK1 (BD Bioscience, cat# 611072; 1:1000 dilution), SRPK2 (BD Bioscience, cat# 611118; 1:1000 dilution), Cleaved-caspase-3 (Cell Signaling Technology, cat#9661; 1:1000 dilution), mAb104 hybridoma (ATCC® CRL-2067™; 1:500 dilution), GAPDH (Santa Cruz, cat#sc-47724; 1:1000 dilution), tubulin (Santa Cruz, cat#sc-23948; 1:1000 dilution), and VEGF_165_b [R&D Systems, cat#MAB3045 and Covalab, in-house antibody; 1:500) [25, 30]]. Immunoblotting was performed as previously described [52]. For total proteins collection, 0.2–0.3 × 10^6^/well HUVEC or HDMEC was plated in 6-well plate with full medium during 24 h, then full medium was removed and cells were refreshed with either 10% EGM-2 full medium for HUVEC or EBM-2 basal medium for HDMEC with or without 1/3 nM FGF-2. In some experiments, spliceosome inhibitors were added at the same time than FGF-2. After 3 days, total proteins were extracted from HUVEC or HDMEC using RIPA 1X buffer (Cell Signaling Technology) supplemented just before use with 1X EDTA-free Protease Inhibitor Cocktail (Roche), 20mM NaF and 1mM Na3VO4. 15–20 μg proteins were loaded onto NuPAGE 4–12% gel (Life Technologies, USA). For analyses of mouse engrafted sub-cutaneous sponges, proteins were isolated from sponges using a 3-min ultrasonic cycle homogenization (cycle of 15 s sonication, 10 s resting time) at around 3–4°C using a circulation water cooling system, followed by a 30-min extraction in ice using RIPA 1X buffer supplemented just before use with protease and phosphate inhibitors. Samples were vortexted for 15 s by every 15 min. After centrifugation for 20 min at 12.000 rpm at 4°C, supernatants were collected, and protein amount was quantified for each sponge using BCA. All sponge samples (6 sponges/each group) were equalized in lysis buffer at a final concentration of 3.5mg/ml. For each group, a pool of 6 sponge samples was done and 30–50 μg proteins were analyzed by immunoblotting after loading on NuPAGE 4-12% gels (Life Technologies, USA). Three independent pooled samples for PBS (*n*=6) and FGF-2 (*n*=6) group were analyzed. The intensity of specific band was measured by ImageJ (NIH software).

### RT-PCR

The primers used in RT-PCR analyses were as follows: VEGFR1: 5′-TCA-GGA-AGC-ACC-ATA-CCT-CC-3′ (foward) and 5′-TGA-ACT-TTC-CAC-AGA-GCC-CTT-3′ (reverse); sVEGFR1-ex15a: 5′-TCA-GGA-AGC-ACC-ATA-CCT-CC-3′ (forward) and 5′-CGT-TGA-TGT-ATA-CAG-TTC-AGG-C-3′ (reverse); sVEGFR1-i13: 5′-TCA-CTC-AGC-GCA-TGG-CAA-TA-3′ (forward) and 5′-CAA-ACG-TGC-ACC-AAG-TCG-G-3′ (reverse); sVEGFR1-ex12: 5′-TCA-CTC-AGC-GCA-TGG-CAA-TA-3′ (forward) and 5′-GAA-GAG-AAG-CTT-GTA-GGT-GGC-3′; GAPDH: 5′-CGA-GAT-CCC-TCC-AAA-ATC-AA-3′ (forward) and 5′-ATC-CAC-AGT-CTT-CTG-GGT-GG-3′ (reverse). The primer’s location is highlighted in Additional File 2: Fig S2. All primers were blasted on NCBI to ensure the selective recognition of the intended VEGFR1 splice variant. For VEGFR1 (variant 1), forward and reverse primers are located on exons 14 and 15, respectively, and PCR amplifies a 260-bp fragment. For sVEGFR1-exon 15a (variant 3), forward and reverse primers are located on exon 14 (same as for VEGFR1) and at the junction between exon 14 and alternative last exon 15 (reverse primer is totally 22bp and 5bp cover exon 14), respectively, and PCR amplifies a 163bp fragment. For sVEGFR1-intron 13 (variant 2), forward and reverse primers are located on exon 11 and 3’UTR, respectively, and PCR amplifies a 589-bp fragment. For sVEGFR1-exon 12 (variant 4), forward and reverse primers are located on exon 11 (same primer as sVEGFR1-intron 13) and alternative last exon 12, respectively, and PCR amplifies a 88-bp fragment. For RNA collection, the same schedule sketches as those used for protein collection were done. The total RNA was extracted using high pure RNA isolation kit (Roche) according to the manufacturer’s instructions. In total, 1 μg of total RNA was subjected to reverse transcription using iScript RT supermix (Bio-Rad). RT reaction (3.5 μl; 2.5 ng/μl cDNA) was then amplified by PCR for a total of 40 cycles using the following conditions: 94°C for 2 min followed by 5 cycles at 94°C for 30 s, 64°C for 30 s, 72°C for 1 min, followed by 5 cycles at 94°C for 30 s, 61°C for 30 s, 72°C for 1 min, followed at 5 cycles of 94°C for 30 s, 59°C for 30 s, 72°C for 1 min, followed by 25 cycles at 94°C for 30 s, 56°C for 30 s, 72°C for 1 min. DNA amplicons were loaded on 2% agarose gels containing UView loading dye (Bio-Rad) and visualized using a ChemiDoc apparatus (Bio-Rad). The intensity of each band was determined using ImageJ (NIH software). The number of biological replicates for each experiment is indicated in the figure legends.

### RT-qPCR

The primers used in RT-qPCR analyses were as follows: SRSF1: 5′-CGC-GAC-GGC-TAT-GAT-TAC-GA-3′ (forward) and 5′-TTT-TCA-GAC-CGC-CTG-GAT-GG-3′ (reverse); SRSF3: 5′-TGG-CTA-CTA-TGG-ACC-ACT-CC-3′ (forward) and 5′-TCT-CGG-ACT-GCA-TCA-GCT-GC-3′ (reverse); SRPK1: 5′-GGG-CAT-CAT-CTG-CTC-AAG-TGG-A-3′ (forward) and 5′-GTC-AGT-GTG-GAT-GAT-ACG-GCA-C-3′ (reverse); VEGF_total_: 5′-CTT-CCT-ACA-GCA-CAA-CAA-AT-3′ (forward) and 5′-GTC-TTG-CTC-TAT-CTT-TCT-TTG-3′ (reverse); VEGF_121_: 5′-ATA-GAG-CAA-GAC-AAG-AAA-AAT-G-3′ (forward) and 5′-ATC-GTT-CTG-TAT-CAG-TCT-TTC-CT-3′ (reverse); VEGF_165_: 5′-AGA-GCA-AGA-CAA-GAA-AAT-CC-3′ (forward) and 5′-TAC-AAA-CAA-ATG-CTT-TCT-CC-3′ (reverse); VEGF_189_: 5′-TAT-AAG-TCC-TGG-AGC-GTT-C-3′ (forward) and 5′-TAC-ACG-TCT-GCG-GAT-CTT-G-3′ (reverse); GAPDH: 5′-CGA-GAT-CCC-TCC-AAA-ATC-AA-3′ (forward) and 5′-ATC-CAC-AGT-CTT-CTG-GGT-GG-3′ (reverse); VEGFR1: 5′-ACC-GAA-TGC-CAC-CTC-CAT-G-3′ (forward) and 5′-AGG-CCT-TGG-GTT-TGC-TGT-C-3′ (reverse); sVEGFR1-ex15a 5′-ACA-CAG-TGG-CCA-TCA-GCA-GTT-3′ (forward) and 5′-CCC-GGC-CAT-TTG-TTA-TTG-TTA-3′ (reverse); sVEGFR1-i13 5′-AGG-GGA-AGA-AAT-CCT-CCA-GA-3′ (forward) and 5′-CAA-CAA-ACA-CAG-AGA-AGG-3′ (reverse). VEGFR1, sVEGFR1-ex15a, and sVEGFR1-i13 primers for qPCR were similar to those we previously used in [26]. For sVEGFR1-ex12, the same forward and reverse primers as for RT-PCR were used. For RNA collection, the same schedule sketches as those used for protein collection were done. The total RNA was extracted using high pure RNA isolation kit (Roche) according to the manufacturer’s instructions. In total, 0.5–1 μg of total RNA was subjected to reverse transcription using iScript RT supermix (Bio-Rad). Quantitative RT-PCR (RT-qPCR) was performed using iTaq® qPCR Universal SYBR Green Supermix (Bio-Rad). qPCR conditions were 95°C for 10 min, followed by 40 cycles at 95°C for 15 s, 60°C for 45 s, then 65°C for 30 s followed by 60 cycles at 65°C for 5 s, 0.5°C/cycle with a ramp of 0.5°C/s. In all experiments, RT-qPCR quantification of the reference gene glyceraldehyde-3-phosphate dehydrogenase (GAPDH) was performed for each sample. The quantification of the expression of each target gene was analyzed as the normalized expression (ΔΔCt) related to GAPDH expression with CFX Maestro Software (Bio-Rad). The fold change (2^-ΔΔCt^) in FGF-2 treated conditions compared to untreated conditions was calculated.

### Quantification of sVEGFR1 by ELISA

ELISA assays were performed in duplicate or triplicate in 96-well plates using a Quantikine sVEGFR1 kit (R&D Systems). Manipulations were carried out according to the manufacturer’s instructions. Briefly, 0.5 × 10^6^ HDMEC/well/1mL were seeded in 6-well plates in EGM-2 full medium overnight, then full medium was removed and washed twice with warm HANKs buffer followed by EBM-2 basal medium culture for 1 day or 3 days in the presence or absence of FGF-2 (3nM) with or without spliceosome inhibitors (e.g., 5 μM SPHINX31 or 10 μM SRPIN340). To reduce evaporation for long time treatment, all well gaps were filled with PBS. After centrifugation at 200g for 10 min at +4°C, all supernatants were collected and stored at −80°C before analysis. The concentration of sVEGFR1 in the supernatants was calculated from the absorbance value compared to the standard curve and expressed in pg/ml.

### Sprouting assay

Analysis of endothelial cells invasion and sprouting in 3D collagen I matrix was performed as previously described [32, 53] with optimization for high magnification and confocal image analysis. In order to analyze the effects of SRSF1, SRSF3, sVEGFR1-i13, or sVEGFR1-ex12 knockdown on FGF-2-induced endothelial cells sprouting, 0.3 × 10^6^ cells/well were seeded overnight in 6-well plates. HUVEC-RFP cells were then subjected to two rounds of transfection at 24 and 48 h using control siRNA or a mixture (50/50) of two distincts siRNA specifically targeting either SRSF1, SRSF3, sVEGFR1-i13, or sVEGFR1-ex12 mRNA. Cells were then trypsinized, washed one time with warm HANKs buffer, and used for 3D invasion assay. In order to test the effects of SRPKs inhibitors on FGF-2-induced endothelial cells sprouting, 0.3 × 10^6^ HUVEC-RFP cells/well were plated in 6-well plates overnight. Cells were then treated with 3nM FGF-2 for 24 h in the presence or absence of 5μM SPHINX31 or 10μM SRPIN340. Cells were then trypsinized, washed once with warm HANKs buffer, and used for 3D invasion assay. Secure-Seal™ Hybridization chambers (ThermoFisher, cat#S24732) adhering to the bottom of labtek (ThermoFisher, cat#155380) were prepared for confocal imaging. Collagen I (Corning, cat#354236, Boulogne Billancourt, France) together with sphingosine 1-phosphate (S1P) and FGF-2 (24nM) were added into the chamber. After 14–18 h, HUVEC-RFP were fixed using 4% PFA and nuclei were counterstained with Hoechst 33342. Confocal image analysis was performed using a confocal microscope LSM710 NLO (Zeiss AxioObserver Z1). HUVEC-RFP 3D invasion ability was quantified manually by counting the number of cells indicated by nucleus number and the invasion distance with the software ZEN 2010 3D projection. The 3D projection for the figures of all the invasion assay was synthesized using either software ZEN 2010 3D projection or a maximun intensity Z-projection by Fiji (NIH software).

### Sponge assay

All mouse animal procedures were conducted following the European Union guidelines (regulation n°86/609), taken in the French law (decree 87/848) regulating animal experimentation and approved by the ethics committee of Grenoble, France (C2EA-12 ComEth Grenoble). Cellulose sponges (thickness 2 mm, diameter 10 mm, HYCAIL Ltd; Turku, Finland) were implanted under the skin of NMRI nude mice as previously described [33]. Operations were performed under general anesthesia induced by intraperitoneal injections of Domitor™ (Pfizer, Orsay, France) and Imalgene™ (Merial, Lyon, France). The mice with sponges were divided into 2 groups of 6 mice (PBS and FGF-2). The sponges were hydrated with 50 μL of PBS 1X or FGF-2 (200 ng/50 μL). PBS 1X or FGF-2 was repeatedly injected into the sponges through the skin on days 1, 2, and 3. At 7 days after implantation, the mice were anesthetized and the sponges were rapidly excised and photographed. Each sponge was then homogenized in 1 mL RIPA lysis buffer with protease and phosphatase inhibitors to extract total proteins from neo-vessels having invaded sponge.

### Fish husbandry, treatment, and immunostaining

Tg(fli1:EGFP) Casper zebrafish maintenance and embryo collection were carried out at the zebrafish PRECI facility (UMS CNRS 3444 Lyon Biosciences, Gerland) in compliance with French Government guidelines (agreement number B693870602). Embryos obtained from natural spawning were raised following standard conditions. Developmental stages are given in hours post-fertilization (hpf) at 28.5°C according to morphological criteria [54]. For drug treatment, Tg(fli1:EGFP) Casper zebrafish embryos were manually dechorionated at 20hpf and placed into individual wells in 96 well plates containing 100μl of E3 medium supplemented with 0.5% DMSO alone or SSR128129E (SSR) (Selleckchem, # NVP-BGJ398) or SRPIN340 diluted in 0.5% DMSO at the indicated concentrations. Embryos were then fixed at 28hpf and 42hpf in 4% paraformaldehyde overnight at 4°C or 4 h at room temperature and transferred in 100% methanol for storage at −20°C until use. Whole-mount immunostaining were performed as previously described [55]. Briefly, embryos were permeabilized in PBS-T (1% Triton X-100 in PBS) and then incubated with proteinase K (Roche) for 20 min. Embryos were incubated overnight at 4°C with rabbit monoclonal anti-GFP (1:100, Clinisciences, # TP401) and then with anti-rabbit IgG coupled to AlexaFluor-488 (1:500, Invitrogen, # ALL034). Embryos were stored at 4°C until observation. Images of embryos from 3 independent experiments were obtained with a Leica SP8 confocal or a Leica DM6000 epifluorescence microscope. Movies were done using the LasX software from LEICA.

### Analysis of TCGA RNA-Seq dataset

FGF-2 and FGFR1 gene expression values as well as sVEGFR1-exon12 usage value were retrieved from publicly available TCGA Lung Squamous Cell Carcinoma (TCGA LUSC) dataset and downloaded using the TCGA Splicing Variants DB database (http://www.tsvdb.com/index.html). FGF-2 and FGFR1 expression values were calculated according to gene expression normalized read counts (log2 RSEM). sVEGFR1-exon12 usage value represents the ratio: exon 12 expression value versus gene expression value. The survival time of LUSC patients were obtained from TCGA Splicing Variants DB as well. Overall survival analysis was performed by stratifying patients into low and high groups according to sVEGFR1 exon 12 usage value and Kaplan-Meier plots were generated by GraphPad Prism 6.0.

### Statistical analysis

The data were analyzed using Graphpad Prism 6.0 software (San Diego, CA). Data were expressed as mean ± SEM or ± SD of independent experiments as indicated in the legends of the figures. The number of experimental replicates and methods of statistical analyses were indicated in the legends of the figures.

## Supplementary Information

**Additional File 1: Figure S1.** Effects of FGFR (AZD4547) and SRPK1 (SRPIN340, SPHINX31) inhibitors on VEGF_165_b protein and total VEGF-A, VEGF_121_, VEGF_165_ and VEGF_189_ mRNA levels in endothelial cells treated or not with FGF-2. (**a**) Representative VEGF_165_b immunoblots in HUVEC and HDMEC treated or not (NT) for 72 hours with 3nM FGF-2 in the presence or absence of 10nM AZD4547 (FGFRinh), 10μM SRPIN340 or 5μM SPHINX31 as indicated. GAPDH was used as a loading control. Representative immunoblots of two (HUVEC) and three (HDMEC) independent experiments are presented. (**b**) HDMEC cells were treated (FGF) or not (NT) with 3nM FGF-2 for 72 hours in the presence or absence of 5μM SPHINX31 (SPH31) or 10μM SRPIN340 as indicated. Graphs represent mean values ± SD of normalized expression of each transcript according to GAPDH mRNA level in 3 independent experiments. For each transcript, the fold change was calculated with value 1 assigned to the normalized expression value obtained in the non treated (control) condition. Unpaired t test, *p<0.05, **p< 0.01, ns: not significant. (PPTX 807 kb)

**Additional File 2:Figure S2.** Schematic representation of VEGFR1 and VEGFR1 splice variants. sVEGFR1-ex15a results from activation of a cryptic 3’-splice acceptor site upon the use of an alternative polyadenylation site in the latter half of intron 14. sVEGFR1-i13 short and sVEGFR1-i13 long result from alternative polyadenylation at different sites in intron 13 to yield mRNAs encoding the same 867 amino acid sVEGFR1 protein isoform, but with either a 17 or 4146 nt 3’-UTR region. sVEGFR1-ex12 retains an alternative last exon (exon 12). The location of forward and reverse primers used in RT-PCR analyses are indicated as black arrows on each transcript. siRNA sequences target exon 12 for sVEGFR1-ex12 and the junction between exon 13 and retained intron 13 for sVEGFR1-i13. (PPTX 68 kb)

## Data Availability

All data generated or analyzed during this study are included in this published article and its supplementary information files. Raw data generated in XCelligence Assay are available from the corresponding author on reasonable request.

## References

[1] Folkman J (2002). Role of angiogenesis in tumor growth and metastasis. Semin Oncol.

[2] Bikfalvi A, Klein S, Pintucci G, Rifkin DB (1997). Biological roles of fibroblast growth factor-2. Endocr Rev..

[3] Ensoli B, Gendelman R, Markham P, Fiorelli V, Colombini S, Raffeld M, Cafaro A, Chang HK, Brady JN, Gallo RC (1994). Synergy between basic fibroblast growth factor and HIV-1 Tat protein in induction of Kaposi's sarcoma. Nature..

[4] Presta M, Dell'Era P, Mitola S, Moroni E, Ronca R, Rusnati M (2005). Fibroblast growth factor/fibroblast growth factor receptor system in angiogenesis. Cytokine Growth Factor Rev.

[5] Klein S, Giancotti FG, Presta M, Albelda SM, Buck CA, Rifkin DB (1993). Basic fibroblast growth factor modulates integrin expression in microvascular endothelial cells. Mol Biol Cell..

[6] Javerzat S, Auguste P, Bikfalvi A (2002). The role of fibroblast growth factors in vascular development. Trends Mol Med..

[7] Murakami M, Nguyen LT, Zhuang ZW, Moodie KL, Carmeliet P, Stan RV (2008). The FGF system has a key role in regulating vascular integrity. J Clin Invest.

[8] Hatanaka K, Lanahan AA, Murakami M, Simons M (2012). Fibroblast growth factor signaling potentiates VE-cadherin stability at adherens junctions by regulating SHP2. PLoS One..

[9] Ribatti D, Vacca A, Roncali L, Dammacco F (2000). The chick embryo chorioallantoic membrane as a model for in vivo research on anti-angiogenesis. Curr Pharm Biotechnol..

[10] Seghezzi G, Patel S, Ren CJ, Gualandris A, Pintucci G, Robbins ES, Shapiro RL, Galloway AC, Rifkin DB, Mignatti P (1998). Fibroblast growth factor-2 (FGF-2) induces vascular endothelial growth factor (VEGF) expression in the endothelial cells of forming capillaries: an autocrine mechanism contributing to angiogenesis. J Cell Biol..

[11] Coltrini D, Di Salle E, Ronca R, Belleri M, Testini C, Presta M (2013). Matrigel plug assay: evaluation of the angiogenic response by reverse transcription-quantitative PCR. Angiogenesis..

[12] De Smet F, Tembuyser B, Lenard A, Claes F, Zhang J, Michielsen C (2014). Fibroblast growth factor signaling affects vascular outgrowth and is required for the maintenance of blood vessel integrity. Chem Biol.

[13] Broadley KN, Aquino AM, Woodward SC, Buckley-Sturrock A, Sato Y, Rifkin DB, Davidson JM (1989). Monospecific antibodies implicate basic fibroblast growth factor in normal wound repair. Lab Invest..

[14] Oladipupo SS, Smith C, Santeford A, Park C, Sene A, Wiley LA, Osei-Owusu P, Hsu J, Zapata N, Liu F, Nakamura R, Lavine KJ, Blumer KJ, Choi K, Apte RS, Ornitz DM (2014). Endothelial cell FGF signaling is required for injury response but not for vascular homeostasis. Proc Natl Acad Sci U S A..

[15] Pan Q, Shai O, Lee LJ, Frey BJ, Blencowe BJ (2008). Deep surveying of alternative splicing complexity in the human transcriptome by high-throughput sequencing. Nat Genet..

[16] Nowak DG, Amin EM, Rennel ES, Hoareau-Aveilla C, Gammons M, Damodoran G, Hagiwara M, Harper SJ, Woolard J, Ladomery MR, Bates DO (2010). Regulation of vascular endothelial growth factor (VEGF) splicing from pro-angiogenic to anti-angiogenic isoforms: a novel therapeutic strategy for angiogenesis. J Biol Chem..

[17] Anczukow O, Krainer AR (2016). Splicing-factor alterations in cancers. RNA..

[18] Nilsen TW, Graveley BR (2010). Expansion of the eukaryotic proteome by alternative splicing. Nature..

[19] Fu XD, Ares M (2014). Context-dependent control of alternative splicing by RNA-binding proteins. Nat Rev Genet..

[20] Sanford JR, Ellis J, Caceres JF (2005). Multiple roles of arginine/serine-rich splicing factors in RNA processing. Biochem Soc Trans.

[21] Lai MC, Lin RI, Tarn WY (2001). Transportin-SR2 mediates nuclear import of phosphorylated SR proteins. Proc Natl Acad Sci U S A..

[22] Zhong XY, Ding JH, Adams JA, Ghosh G, Fu XD (2009). Regulation of SR protein phosphorylation and alternative splicing by modulating kinetic interactions of SRPK1 with molecular chaperones. Genes Dev..

[23] Gout S, Brambilla E, Boudria A, Drissi R, Lantuejoul S, Gazzeri S, Eymin B (2012). Abnormal expression of the pre-mRNA splicing regulators SRSF1, SRSF2, SRPK1 and SRPK2 in non small cell lung carcinoma. PLoS One..

[24] Nowak DG, Woolard J, Amin EM, Konopatskaya O, Saleem MA, Churchill AJ, Ladomery MR, Harper SJ, Bates DO (2008). Expression of pro- and anti-angiogenic isoforms of VEGF is differentially regulated by splicing and growth factors. J Cell Sci.

[25] Merdzhanova G, Gout S, Keramidas M, Edmond V, Coll JL, Brambilla C, Brambilla E, Gazzeri S, Eymin B (2010). The transcription factor E2F1 and the SR protein SC35 control the ratio of pro-angiogenic versus antiangiogenic isoforms of vascular endothelial growth factor-A to inhibit neovascularization in vivo. Oncogene..

[26] Abou Faycal C, Gazzeri S, Eymin B (2019). A VEGF-A/SOX2/SRSF2 network controls VEGFR1 pre-mRNA alternative splicing in lung carcinoma cells. Sci Rep..

[27] Amin EM, Oltean S, Hua J, Gammons MV, Hamdollah-Zadeh M, Welsh GI (2011). WT1 mutants reveal SRPK1 to be a downstream angiogenesis target by altering VEGF splicing. Cancer Cell.

[28] Gammons MV, Lucas R, Dean R, Coupland SE, Oltean S, Bates DO (2014). Targeting SRPK1 to control VEGF-mediated tumour angiogenesis in metastatic melanoma. Br J Cancer..

[29] Batson J, Toop HD, Redondo C, Babaei-Jadidi R, Chaikuad A, Wearmouth SF, Gibbons B, Allen C, Tallant C, Zhang J, du C, Hancox JC, Hawtrey T, da Rocha J, Griffith R, Knapp S, Bates DO, Morris JC (2017). Development of potent, selective srpk1 inhibitors as potential topical therapeutics for neovascular eye disease. ACS Chem Biol..

[30] Boudria A, Abou Faycal C, Jia T, Gout S, Keramidas M, Didier C, Lemaître N, Manet S, Coll JL, Toffart AC, Moro-Sibilot D, Albiges-Rizo C, Josserand V, Faurobert E, Brambilla C, Brambilla E, Gazzeri S, Eymin B (2019). VEGF165b, a splice variant of VEGF-A, promotes lung tumor progression and escape from anti-angiogenic therapies through a beta1 integrin/VEGFR autocrine loop. Oncogene.

[31] Siqueira RP, Barbosa Ede A, Poleto MD, Righetto GL, Seraphim TV, Salgado RL (2015). Potential antileukemia effect and structural analyses of SRPK inhibition by N-(2-(piperidin-1-yl)-5-(trifluoromethyl)phenyl)isonicotinamide (SRPIN340). PLoS One.

[32] Bayless KJ, Kwak HI, Su SC (2009). Investigating endothelial invasion and sprouting behavior in three-dimensional collagen matrices. Nat Protoc..

[33] Keramidas M, Josserand V, Feige JJ, Coll JL (2013). Noninvasive and quantitative assessment of in vivo angiogenesis using RGD-based fluorescence imaging of subcutaneous sponges. Mol Imaging Biol..

[34] Saito T, Takeda N, Amiya E, Nakao T, Abe H, Semba H, Soma K, Koyama K, Hosoya Y, Imai Y, Isagawa T, Watanabe M, Manabe I, Komuro I, Nagai R, Maemura K (2013). VEGF-A induces its negative regulator, soluble form of VEGFR-1, by modulating its alternative splicing. FEBS Lett..

[35] Heydarian M, McCaffrey T, Florea L, Yang Z, Ross MM, Zhou W, Maynard SE (2009). Novel splice variants of sFlt1 are upregulated in preeclampsia. Placenta..

[36] Ashar-Patel A, Kaymaz Y, Rajakumar A, Bailey JA, Karumanchi SA, Moore MJ (2017). FLT1 and transcriptome-wide polyadenylation site (PAS) analysis in preeclampsia. Sci Rep..

[37] Inoue T, Kibata K, Suzuki M, Nakamura S, Motoda R, Orita K (2000). Identification of a vascular endothelial growth factor (VEGF) antagonist, sFlt-1, from a human hematopoietic cell line NALM-16. FEBS Lett..

[38] Orecchia A, Lacal PM, Schietroma C, Morea V, Zambruno G, Failla CM (2003). Vascular endothelial growth factor receptor-1 is deposited in the extracellular matrix by endothelial cells and is a ligand for the alpha 5 beta 1 integrin. J Cell Sci.

[39] Soro S, Orecchia A, Morbidelli L, Lacal PM, Morea V, Ballmer-Hofer K, Ruffini F, Ziche M, D'Atri S, Zambruno G, Tramontano A, Failla CM (2008). A proangiogenic peptide derived from vascular endothelial growth factor receptor-1 acts through alpha5beta1 integrin. Blood..

[40] Giacomini A, Chiodelli P, Matarazzo S, Rusnati M, Presta M, Ronca R (2016). Blocking the FGF/FGFR system as a “two-compartment” antiangiogenic/antitumor approach in cancer therapy. Pharmacol Res..

[41] Fujii T, Kuwano H (2010). Regulation of the expression balance of angiopoietin-1 and angiopoietin-2 by Shh and FGF-2. In Vitro Cell Dev Biol Anim..

[42] Murakami M, Simons M (2008). Fibroblast growth factor regulation of neovascularization. Curr Opin Hematol..

[43] Mallinjoud P, Villemin JP, Mortada H, Polay Espinoza M, Desmet FO, Samaan S, Chautard E, Tranchevent LC, Auboeuf D (2014). Endothelial, epithelial, and fibroblast cells exhibit specific splicing programs independently of their tissue of origin. Genome Res..

[44] Wagner KD, El Mai M, Ladomery M, Belali T, Leccia N, Michiels JF, et al. Altered VEGF splicing isoform balance in tumor endothelium involves activation of splicing factors Srpk1 and Srsf1 by the Wilms’ tumor suppressor Wt1. Cells. 2019;8(1). 10.3390/cells8010041. 10.3390/cells8010041 PMC6356959 30641926

[45] Eisenreich A, Bogdanov VY, Zakrzewicz A, Pries A, Antoniak S, Poller W, Schultheiss HP, Rauch U (2009). Cdc2-like kinases and DNA topoisomerase I regulate alternative splicing of tissue factor in human endothelial cells. Circ Res..

[46] Kearney JB, Ambler CA, Monaco KA, Johnson N, Rapoport RG, Bautch VL (2002). Vascular endothelial growth factor receptor Flt-1 negatively regulates developmental blood vessel formation by modulating endothelial cell division. Blood..

[47] Chappell JC, Taylor SM, Ferrara N, Bautch VL (2009). Local guidance of emerging vessel sprouts requires soluble Flt-1. Dev Cell..

[48] Orecchia A, Mettouchi A, Uva P, Simon GC, Arcelli D, Avitabile S, Ragone G, Meneguzzi G, Pfenninger KH, Zambruno G, Failla CM (2014). Endothelial cell adhesion to soluble vascular endothelial growth factor receptor-1 triggers a cell dynamic and angiogenic phenotype. FASEB J..

[49] Weeden CE, Solomon B, Asselin-Labat ML (2015). FGFR1 inhibition in lung squamous cell carcinoma: questions and controversies. Cell Death Discov..

[50] Hashemi-Sadraei N, Hanna N (2017). Targeting FGFR in squamous cell carcinoma of the lung. Target Oncol..

[51] Song Y, Li L, Ou Y, Gao Z, Li E, Li X, Zhang W, Wang J, Xu L, Zhou Y, Ma X, Liu L, Zhao Z, Huang X, Fan J, Dong L, Chen G, Ma L, Yang J, Chen L, He M, Li M, Zhuang X, Huang K, Qiu K, Yin G, Guo G, Feng Q, Chen P, Wu Z, Wu J, Ma L, Zhao J, Luo L, Fu M, Xu B, Chen B, Li Y, Tong T, Wang M, Liu Z, Lin D, Zhang X, Yang H, Wang J, Zhan Q (2014). Identification of genomic alterations in oesophageal squamous cell cancer. Nature..

[52] Jia T, Choi J, Ciccione J, Henry M, Mehdi A, Martinez J, Eymin B, Subra G, Coll JL (2018). Heteromultivalent targeting of integrin alphavbeta3 and neuropilin 1 promotes cell survival via the activation of the IGF-1/insulin receptors. Biomaterials.

[53] Jia T, Vaganay E, Carpentier G, Coudert P, Guzman-Gonzales V, Manuel R, Eymin B, Coll JL, Ruggiero F (2020). A collagen Valpha1-derived fragment inhibits FGF-2 induced-angiogenesis by modulating endothelial cells plasticity through its heparin-binding site. Matrix Biol.

[54] Kimmel CB, Ballard WW, Kimmel SR, Ullmann B, Schilling TF (1995). Stages of embryonic development of the zebrafish. Dev Dyn..

[55] Guillon E, Bretaud S, Ruggiero F (2016). Slow muscle precursors lay down a collagen XV matrix fingerprint to guide motor axon navigation. J Neurosci..

