# Supplementary material for: FGF-2 promotes angiogenesis through a SRSF1/SRSF3/SRPK1-dependent axis that controls VEGFR1 splicing in endothelial cells
Source: BMC Biol. 2021 Aug 25;19:173. doi: 10.1186/s12915-021-01103-3 (PMC8390225; doi:10.1186/s12915-021-01103-3)
Supplement: Supplementary file 7 — Additional File 1: Figure S1. Effects of FGFR (AZD4547) and SRPK1 (SRPIN340, SPHINX31) inhibitors on VEGF165b protein and total VEGF-A, VEGF121, VEGF165 and VEGF189 mRNA levels in endothelial cells treated or not with FGF-2. (a) Representative VEGF165b immunoblots in HUVEC and HDMEC treated or not (NT) for 72 hours with 3nM FGF-2 in the presence or absence of 10nM AZD4547 (FGFRinh), 10μM SRPIN340 or 5μM SPHINX31 as indicated. GAPDH was used as a loading control. Representative immunoblots of two (HUVEC) and three (HDMEC) independent experiments are presented. (b) HDMEC cells were treated (FGF) or not (NT) with 3nM FGF-2 for 72 hours in the presence or absence of 5μM SPHINX31 (SPH31) or 10μM SRPIN340 as indicated. Graphs represent mean values ± SD of normalized expression of each transcript according to GAPDH mRNA level in 3 independent experiments. For each transcript, the fold change was calculated with value 1 assigned to the normalized expression value obtained in the non treated (control) condition. Unpaired t test, *p<0.05, **p< 0.01, ns: not significant. (PPTX 807 kb) [file 12915_2021_1103_MOESM1_ESM.pptx]

## Slide 1
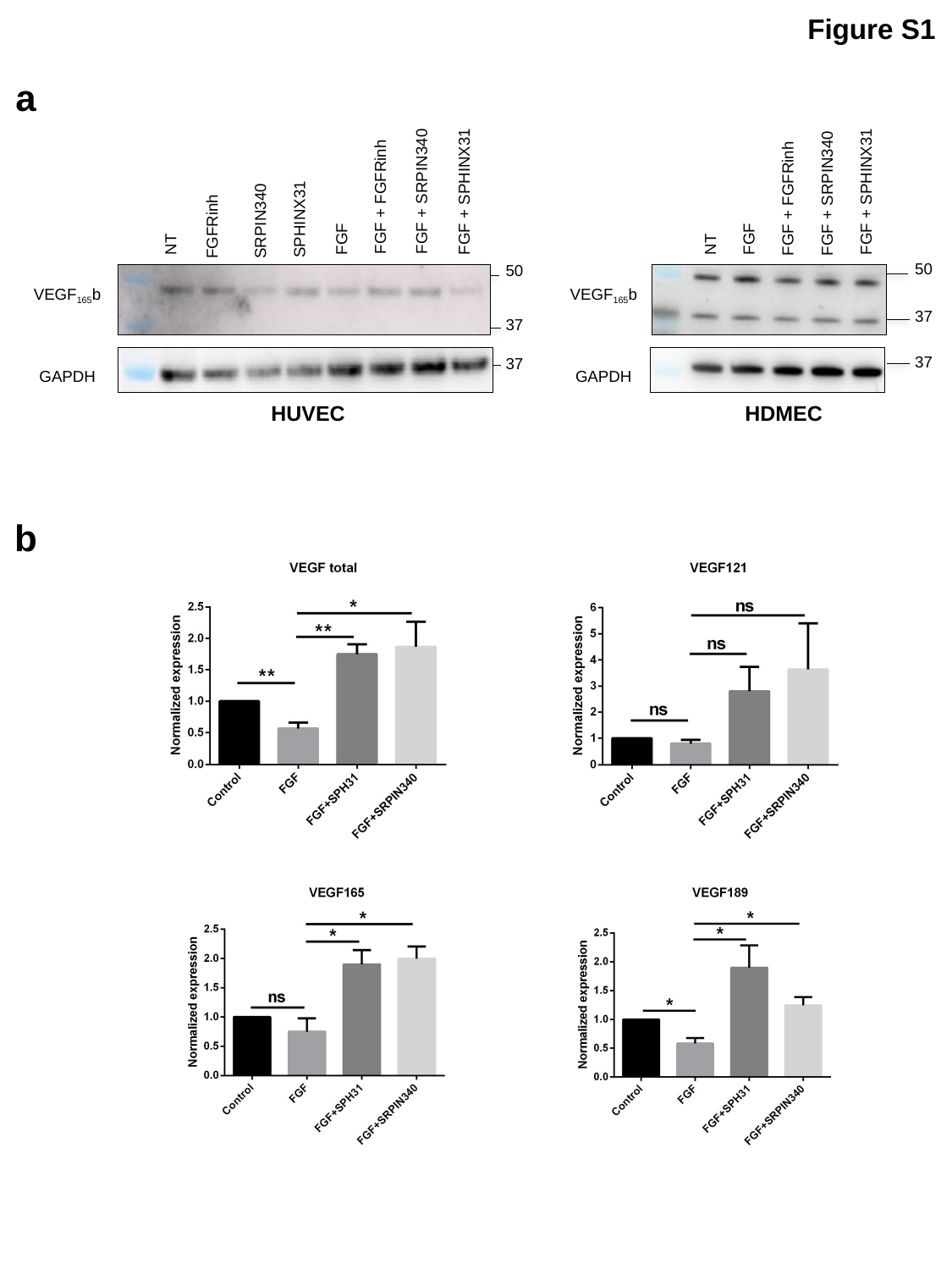

Figure S1
a
FGF + SPHINX31
FGF + SRPIN340
FGF + FGFRinh
FGF
NT
50
VEGF165b
37
37
GAPDH
HDMEC
FGF + SRPIN340
FGF + SPHINX31
FGF + FGFRinh
SPHINX31
SRPIN340
FGFRinh
FGF
NT
50
VEGF165b
37
37
GAPDH
HUVEC
b
