# Supplementary material for: FGF-2 promotes angiogenesis through a SRSF1/SRSF3/SRPK1-dependent axis that controls VEGFR1 splicing in endothelial cells
Source: BMC Biol. 2021 Aug 25;19:173. doi: 10.1186/s12915-021-01103-3 (PMC8390225; doi:10.1186/s12915-021-01103-3)
Supplement: Supplementary file 8 — Additional File 2:Figure S2. Schematic representation of VEGFR1 and VEGFR1 splice variants. sVEGFR1-ex15a results from activation of a cryptic 3’-splice acceptor site upon the use of an alternative polyadenylation site in the latter half of intron 14. sVEGFR1-i13 short and sVEGFR1-i13 long result from alternative polyadenylation at different sites in intron 13 to yield mRNAs encoding the same 867 amino acid sVEGFR1 protein isoform, but with either a 17 or 4146 nt 3’-UTR region. sVEGFR1-ex12 retains an alternative last exon (exon 12). The location of forward and reverse primers used in RT-PCR analyses are indicated as black arrows on each transcript. siRNA sequences target exon 12 for sVEGFR1-ex12 and the junction between exon 13 and retained intron 13 for sVEGFR1-i13. (PPTX 68 kb) [file 12915_2021_1103_MOESM2_ESM.pptx]

## Slide 1
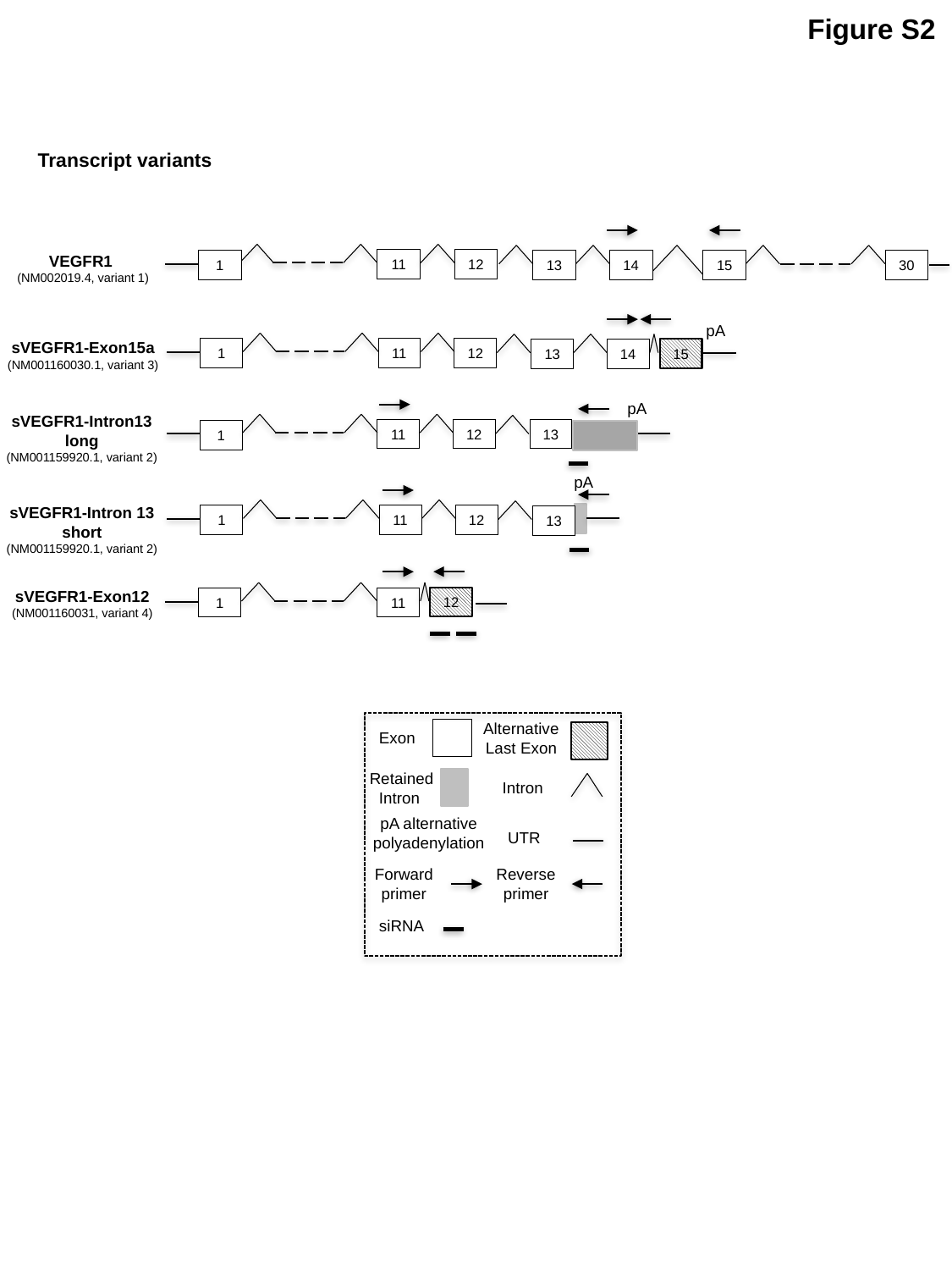

Figure S2
Transcript variants
11
12
13
14
15
30
VEGFR1
(NM002019.4, variant 1)
1
pA
11
12
15
13
14
sVEGFR1-Exon15a
(NM001160030.1, variant 3)
1
pA
sVEGFR1-Intron13 long
(NM001159920.1, variant 2)
11
12
13
1
pA
11
12
sVEGFR1-Intron 13 short
(NM001159920.1, variant 2)
13
1
12
11
sVEGFR1-Exon12
(NM001160031, variant 4)
1
Alternative Last Exon
Exon
Retained Intron
Intron
pA alternative polyadenylation
UTR
Forward primer
Reverse
primer
siRNA
